# Association of tirzepatide with changes in OSA-related measures based on baseline characteristics – post hoc analyses of SURMOUNT-OSA

**DOI:** 10.1007/s44470-026-00162-z

**Published:** 2026-08-31

**Authors:** Beverly Falcon, Cathy Chang Xie, Susan Redline, Ronald Grunstein, Christopher D. Turnbull, David M. Rapoport, Hui Wang, Sujatro Chakladar, Georgios K. Dimitriadis, Eva Lau, Jo Bednarik, Birong Liao, Atul Malhotra

**Affiliations:** 1 Eli Lilly and Company, Global Medical Affairs, Indianapolis, IN 46285 USA; 2 Department of Medicine, Division of Sleep and Circadian Disorders, Brigham and Women’s Hospital, Division of Sleep Medicine, Harvard Medical School, Boston, MA USA; 3 Sleep and Circadian Group, Woolcock Institute of Medical Research, Macquarie University, Royal Prince Alfred Hospital and University of Sydney, Camperdown, NSW Australia; 4 Nuffield Department of Medicine, University of Oxford, Oxford, UK; 5 Icahn School of Medicine at Mount Sinai, New York, NY USA; 6 Techdata Service, LLC, King of Prussia, PA USA; 7 Department of Medicine, UC San Diego Health, San Diego, CA USA

**Keywords:** Obstructive sleep apnea, Obesity, Tirzepatide, Lung, Respiration

## Abstract

**Purpose:**

Obstructive sleep apnea (OSA) is a common disorder characterized by repetitive collapse of the upper airway during sleep. Given that excess adiposity is a known risk factor for OSA, we aimed to descriptively assess the association of tirzepatide, a GIP/GLP-1 receptor agonist, with changes in AHI, hypoxic burden, body weight, and blood pressure in different patient populations based on baseline characteristics such as age, sex, BMI, AHI, and neck circumference.

**Methods:**

These post hoc analyses examined data from two Phase 3 randomized, double-blind studies evaluating maximum tolerated dose (MTD) tirzepatide (10 mg or 15 mg) compared with placebo in adults with moderate-to-severe OSA (AHI ≥ 15 events/h) and obesity (BMI ≥ 30 kg/m^2^) over a 52-week period. Baseline subgroup analyses were conducted in participants with non-missing relevant baseline measurements.

**Results:**

Generally, participants treated with tirzepatide showed greater improvements in OSA outcomes compared with placebo, regardless of baseline subgroup. Participants treated with tirzepatide experienced reductions in AHI across subgroups, regardless of baseline age (-27.7 to -34.1 events/h), sex (-19.8 to -32.6 events/h), AHI severity (-12.1 to -52.2 events/h), BMI (-25.2 to -34.4 events/h), and neck circumference (-23.9 to -30.8 events/h). Additionally, improvements were observed in body weight, systolic blood pressure, and sleep apnea-specific hypoxic burden across baseline subgroups. Overall, most participants experienced an improvement in AHI severity category with tirzepatide treatment (68% to 79%), while the majority in the placebo group saw no clinically relevant change (64% to 70%).

**Conclusions:**

In these descriptive, hypothesis-generating, post hoc analyses, tirzepatide treatment was associated with improvements in multiple measures in participants with moderate-to-severe OSA and obesity. These improvements were observed across both studies, regardless of baseline age, sex, AHI severity, BMI, or neck circumference.

**Clinical Trial Registration:**

SURMOUNT-OSA program (NCT05412004).

**Brief summary:**

**Current knowledge/study rationale:**

Tirzepatide has been associated with clinically relevant improvements in OSA-related measures, body weight, and systolic blood pressure among individuals with moderate-to-severe OSA and obesity. These post hoc analyses aimed to assess whether there were variations in improvements based on baseline age, sex, AHI severity, BMI, or neck circumference.

**Study impact:**

In general, tirzepatide treatment was associated with improvement in OSA outcomes across both studies, regardless of baseline age, sex, AHI severity, BMI, or neck circumference, with some observed differences among some baseline characteristics. This research may help us better understand the relationship between baseline characteristics and different OSA treatment responses and may stimulate future studies in this area.

**Graphical Abstract:**

**Figure:**
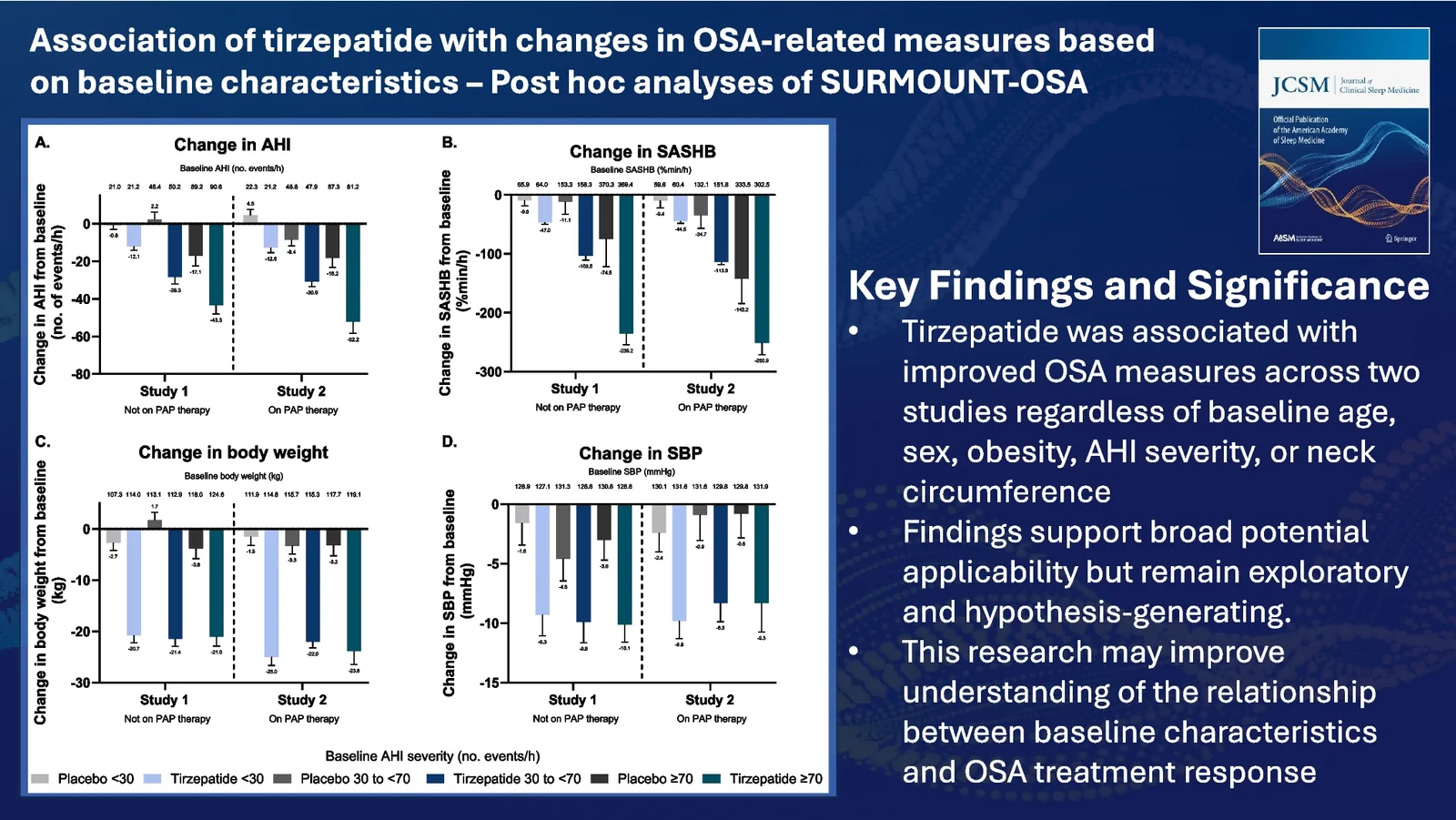

**Supplementary Information:**

The online version contains supplementary material available at https://doi.org/10.1007/s44470-026-00162-z.

## Introduction

Obstructive sleep apnea (OSA) is a common disorder characterized by repetitive collapse of the upper airway during sleep [1]. The intermittent hypoxemia, sleep fragmentation, and intrathoracic pressure swings associated with OSA contribute to adverse outcomes such as excessive daytime sleepiness, cardiovascular risk, and metabolic disease [2].

Obesity, defined by excess adiposity [3], is a global health issue affecting over 890 million people worldwide [4] and is associated with over 200 related complications, [5, 6] increased risk for chronic diseases such as cardiovascular diseases and type 2 diabetes, [7] and increased all-cause mortality [8, 9]. Excess adiposity is a well-established risk factor for OSA [10, 11]. While OSA occurs in individuals without obesity, the risk of developing OSA increases with higher obesity classes [12]. A 10% increase in weight is associated with a sixfold increase in the odds of developing moderate-to-severe OSA [13]. Clinical guidance advises weight reduction in all people with OSA and obesity or overweight [11] and is considered a core component of OSA treatment [14].

Tirzepatide is a once-weekly, dual glucose-dependent insulinotropic polypeptide (GIP) and glucagon-like peptide-1 (GLP-1) receptor agonist approved in some countries for adults with obesity, type 2 diabetes, and moderate-to-severe OSA in people with obesity [15, 16]. The SURMOUNT-OSA program (NCT05412004) included two randomized, placebo-controlled Phase 3 studies that investigated the efficacy and safety of tirzepatide in participants with moderate-to-severe OSA and obesity. Study 1 included participants not on Positive Airway Pressure (PAP) therapy. Study 2 included participants on PAP therapy [17]. These studies showed clinically relevant improvements in the apnea hypopnea index (AHI) and sleep apnea-specific hypoxic burden (SASHB) as markers of sleep apnea severity, as well as systolic blood pressure, body weight (BW), high-sensitivity C-reactive protein, and patient-reported outcomes [17].

There have been limited studies on the effects of new-generation obesity management medications, such as GIP/GLP-1 receptor agonists, on OSA across individuals with different degrees of disease severity and obesity. Tirzepatide is the first pharmacological therapy approved for the treatment of moderate-to-severe OSA in adults with obesity in some countries. It is important to understand better the effects of tirzepatide in different patient populations, given that the mechanisms for OSA vary by demographic factors such as age and sex, as well as obesity [18]. Such studies may also identify differences in weight reduction and current therapy adaptation across populations subgroups. The objective of these current exploratory post hoc analyses was to assess descriptively the association of tirzepatide with changes in OSA measures, BW reduction, and other measures in patient populations characterized by demographic (age and sex), disease severity (AHI), and anthropometric (BMI, neck circumference) features measured before intervention.

## Methods

### Study design and participants

These post hoc analyses examined data from two Phase 3 randomized, double-blind studies. These studies evaluated the efficacy and safety of the MTD tirzepatide (10 mg or 15 mg) against placebo in adults with moderate-to-severe OSA (AHI ≥ 15 events/h) and obesity (BMI ≥ 30 kg/m^2^) over a 52-week period. Study 1 included participants who were not on PAP, while Study 2 included participants who were on PAP. AHI was determined by in-laboratory polysomnograms, whereby hypopneas were determined using the American Academy of Sleep Medicine rule 1B (a ≥ 30% reduction in airflow for ≥ 10 s, and oxygen desaturation of ≥ 4%) [17]. In total, Study 1 included 234 participants (tirzepatide = 114, placebo = 120), while Study 2 had 235 participants (tirzepatide = 120, placebo = 115) [17]. Overall, tirzepatide was generally well-tolerated, with 85% to 90% of participants receiving tirzepatide completing the study (placebo = 70% to 74%) [10]. Full study details and design are available in previously published papers [10, 17].

The SURMOUNT-OSA trials were conducted in accordance with consensus ethical principles, including the Declaration of Helsinki and Council for International Organizations of Medical Sciences International Ethical Guidelines, applicable International Council for Harmonisation Good Clinical Practice guidelines, and applicable laws and regulations, and were approved by the relevant ethics committee/review board at each site. All participants in all primary trials provided written informed consent. The SURMOUNT-OSA program was registered with ClinicalTrials.gov (NCT05412004).

### Baseline subgroup measures

These post hoc analyses assessed different subgroups, based on baseline characteristics of participants from the SURMOUNT-OSA trials. The subgroups assessed were either predefined in the protocol, based on class or severity, or were based on approximately evenly weighted numbers of participants in each subgroup. Subgroups included age, sex, AHI, BMI, and neck circumference, with cut-offs and N values included in Table S1.

### Outcomes

Categorical shifts in OSA severity from baseline severities of moderate OSA (AHI >  = 15- < 30/h) or severe OSA (AHI >  = 30/h) to Week 52 categories of no OSA (AHI < 5/h), mild OSA (AHI >  = 5- < 15/h), moderate OSA, or severe OSA were assessed in participants who had both baseline and Week 52 data. These categorical shifts were classified as Improvement (shift to an improved OSA severity category), No change (remained in the same severity category), or Worsening (shift to a worse OSA severity category). The No change category and the worsening category were combined for baseline subgroup analyses due to their low rates of occurrence.

### Statistical analysis

These post hoc analyses were conducted in the modified intent-to-treat (mITT) population, comprising all randomly assigned participants who received at least one dose of the study intervention, using the efficacy analysis set. The efficacy analysis set specifies the data for each participant in the mITT population that was used in the analyses. Participants who discontinued treatment were included in the analysis, however, data collected after discontinuation were excluded. Additionally, for all the subgroup analyses, only participants with non-missing relevant baseline measurements used to define the subgroups are included. The number of participants with missing baseline data is given in Table S18.

Within each baseline subgroup, model-based estimates (MBE) of change or percent change from baseline at Week 52 for the tirzepatide and placebo arms, and the MBE difference between tirzepatide and placebo, were derived using a mixed model for repeated measures (MMRM). The MMRM controlled for baseline value of the endpoint, geographic region, baseline OSA severity subgroup (for non-AHI-related endpoints), sex (for non-sex-based baseline subgroups), and treatment. Additional fixed effects included visit (representing pre-specified assessment timepoints per the study protocol) and the treatment-by-visit interaction. A log transformation was applied to SASHB prior to fitting the MMRM model. Some assessments of confounding baseline characteristics were performed and demonstrated a consistent direction and magnitude of the treatment effect across subgroups.

Categorical shifts in OSA severity from baseline to Week 52 were evaluated in participants within the efficacy analysis set who had non-missing AHI values at baseline and Week 52. For binary endpoints, missing continuous endpoint values used to derive the binary outcome at Week 52 were imputed using multiple imputation. Then, proportions of participants achieving the endpoint of interest were summarized within each baseline subgroup. In addition, odds ratios between tirzepatide and placebo arms were estimated from logistic regression models, after adjusting for baseline AHI (in non-AHI-related subgroups), geographic region, sex (in non-sex-based baseline subgroups), and treatment, as previously defined in the study protocols [10].

These analyses are descriptive and hypothesis-generating. As such, no formal statistical testing for heterogeneity or treatment effect across subgroups was reported. Apparent subgroup differences may reflect chance, residual confounding, differing baseline distributions, or regression to the mean, and should be interpreted with caution. Differences between subgroups were assessed by the presence or absence of a consistent trend at two levels: 1) if the two studies show the same trend across the levels of a subgroup measure for a given outcome (for example, treatment effect of a given outcome is numerically greater in younger participants than in older participants in both studies), or 2) if the two studies show the same trend across the levels of a subgroup measure for multiple outcomes (for example, treatment effects of both OSA outcomes are numerically greater in younger participants than in older participants in both studies).

## Results

Previous analyses of the full study population focused on treatment regimen analyses [17]. Efficacy analysis of the total study population is included in Supplemental Table 2.

### Age

**Fig. 1:**
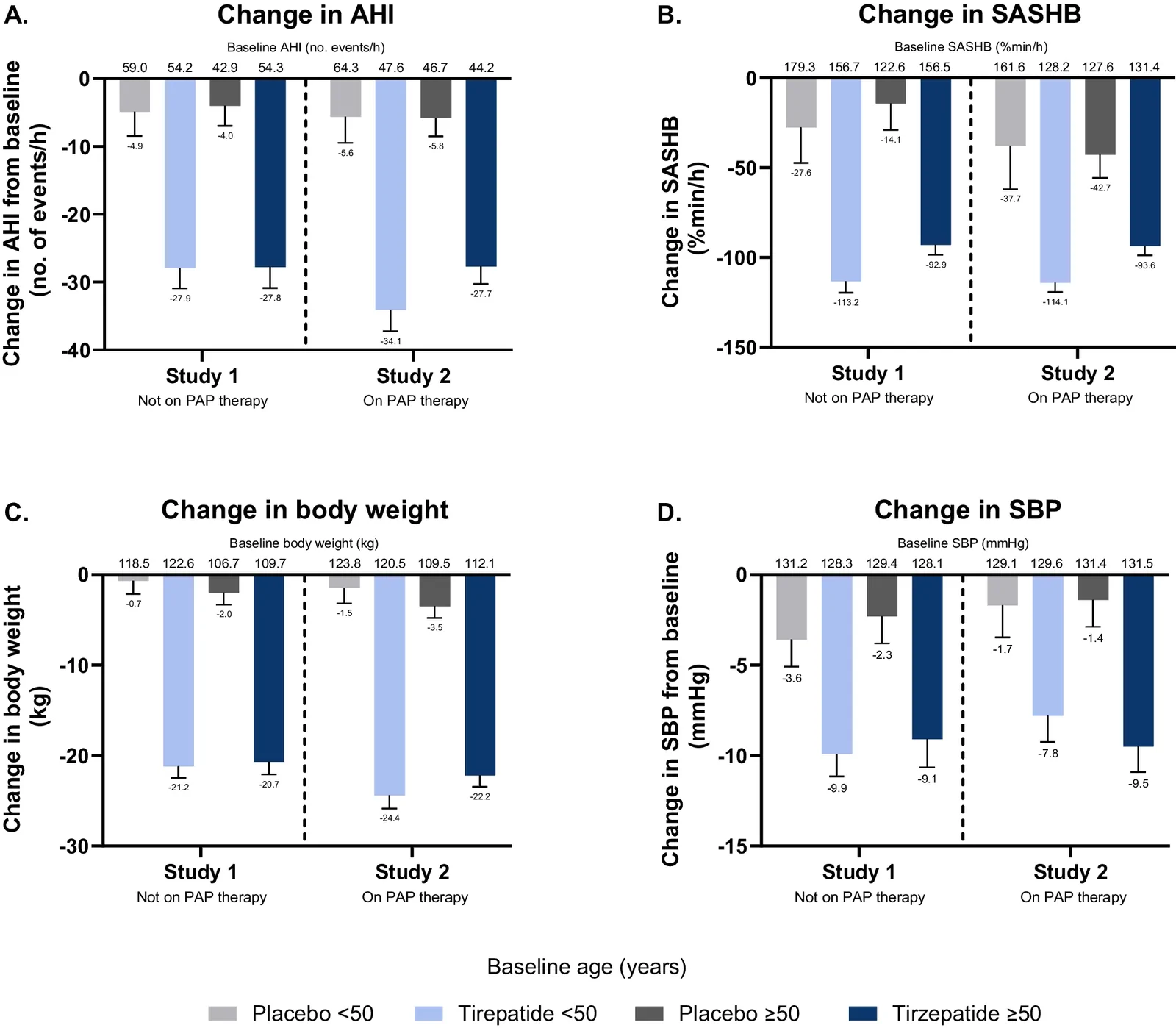
Subgroup age. Absolute changes from baseline in participants receiving tirzepatide or placebo in the SURMOUNT-OSA trial by age group in the following measures: **A**. Change in Apnea Hypopnea Index (AHI), **B**. Change in sleep apnea hypoxic burden (SASHB), **C**. Change in body weight (BW), **D**. Change in systolic blood pressure (SBP). Note: Data are model-based estimates with absolute changes listed below the bars and percent changes from baseline listed within or beside the bars, where available

In the SURMOUNT-OSA trial, there were more females in the older age subgroup (≥ 50 years) compared with the younger age subgroup (< 50 years) in both studies. In addition, AHI, SASHB, BMI, and weight were all lower in the older age group (Table S3). In both studies, tirzepatide treatment was associated with a greater reduction in AHI and SASHB than placebo, regardless of baseline age (Fig. 1A, B, Table S8-9). Participants treated with tirzepatide experienced greater reductions in BW and systolic blood pressure (SBP) compared with placebo, regardless of age subgroup, in each study (Fig. 1C, D, Table S8-9).

Generally, outcomes were similar across age subgroups in both studies. However, in participants treated with tirzepatide in Study 2, numerically greater absolute changes from baseline to Week 52 were observed in the younger age subgroup for AHI (−34.1 events/h in < 50 yrs versus −27.7 events/h in ≥ 50 yrs), SASHB (−114.1%min/h in < 50 yrs versus −93.6%min/h in ≥ 50yrs), and BW (−24.4 kg in < 50 yrs versus −22.2 kg in ≥ 50 yrs), as seen in Table S9. The change in SBP was greater in the older population (Study 2: −7.8 mmHg in < 50 yrs and −9.5 mmHg in ≥ 50yrs, Table S9). Whether these differences reflect true age-related modification of treatment effect or chance cannot be determined from these analyses. In Study 1, younger participants demonstrated similar or slightly smaller treatment differences in AHI, SASHB, BW, and SBP (Table S8).

### Sex

**Fig. 2:**
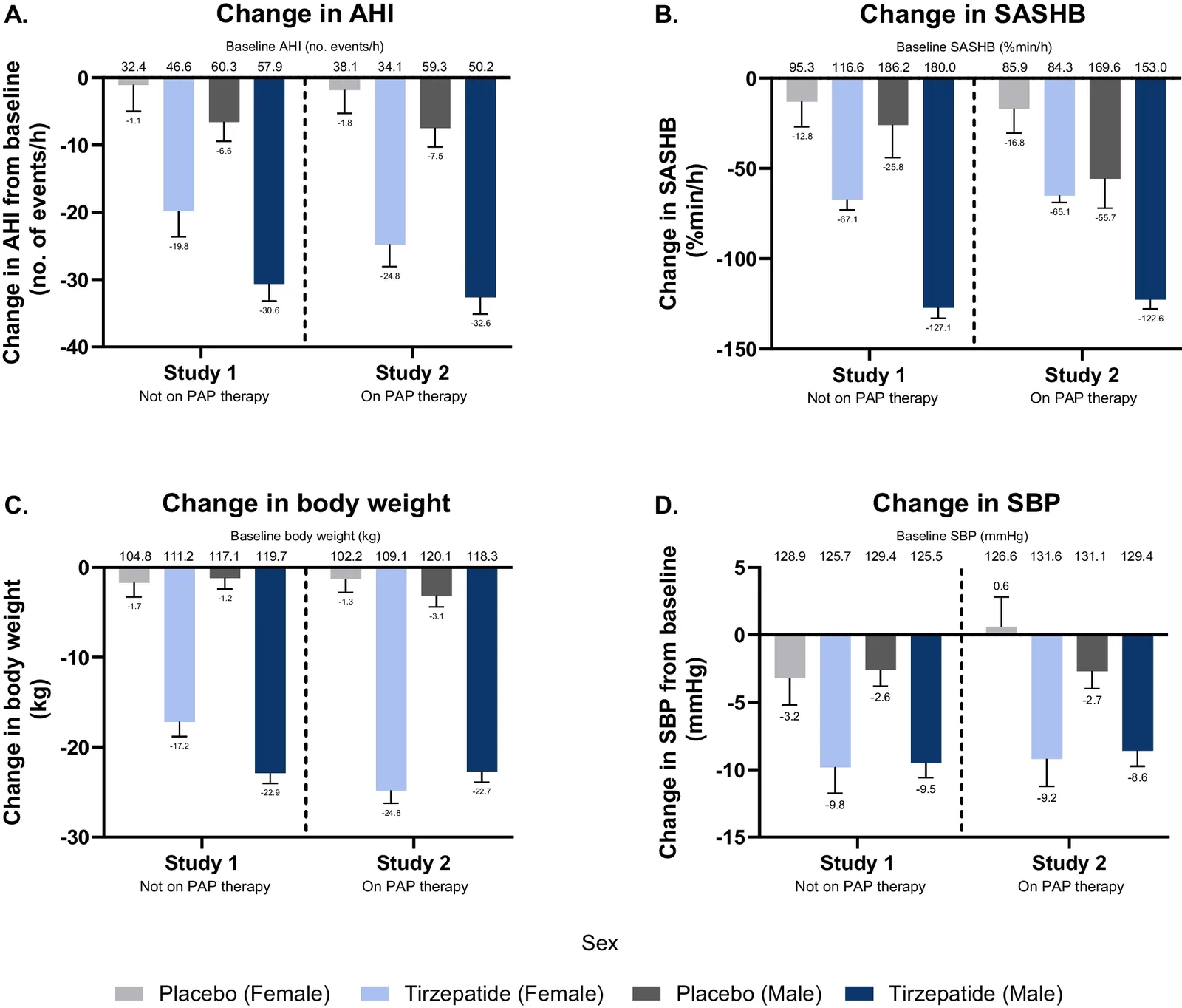
Subgroup sex. Absolute changes from baseline in participants receiving tirzepatide or placebo in the SURMOUNT-OSA trial by sex in the following measures: **A**. Change in Apnea Hypopnea Index (AHI), **B**. Change in sleep apnea hypoxic burden (SASHB), **C**. Change in body weight (BW), **D**. Change in systolic blood pressure (SBP). Note: Data are model-based estimates with absolute changes listed below the bars and percent changes from baseline listed within or beside the bars, where available

In both Study 1 and Study 2, males had a higher mean AHI, SASHB, and BW at baseline but a lower BMI and age compared with females (Table S4). In both studies, tirzepatide treatment was associated with a greater reduction in AHI, SASHB, and BW compared with placebo for males and females (Fig. 2A, B, C, Table S10-11). Additionally, SBP was reduced across both sex subgroups in the tirzepatide arm compared with placebo in both studies (Fig. 2D, Table S10-11).

In general, both females and males showed greater reductions with tirzepatide than with placebo across the demonstrated measures. Absolute reductions in AHI were numerically greater in males treated with tirzepatide than in females in both studies (Study 1: females −19.8 events/h, males −30.6 events/h; Study 2: females −24.8 events/h, males −32.6 events/h; Table S10-11), perhaps due to a higher baseline AHI in males. Similarly, absolute reductions and percent change in SASHB were numerically greater in males than females in both studies, perhaps due to a higher baseline SASHB in males. Numerically greater reductions in BW were observed in females in Study 2 (females: −24.8 kg, males: −22.7 kg), but in males in Study 1 (females: −17.2 kg, males: −22.9 kg; Table S10-11). SBP reductions were broadly similar between sexes across both studies.

### AHI

**Fig. 3:**
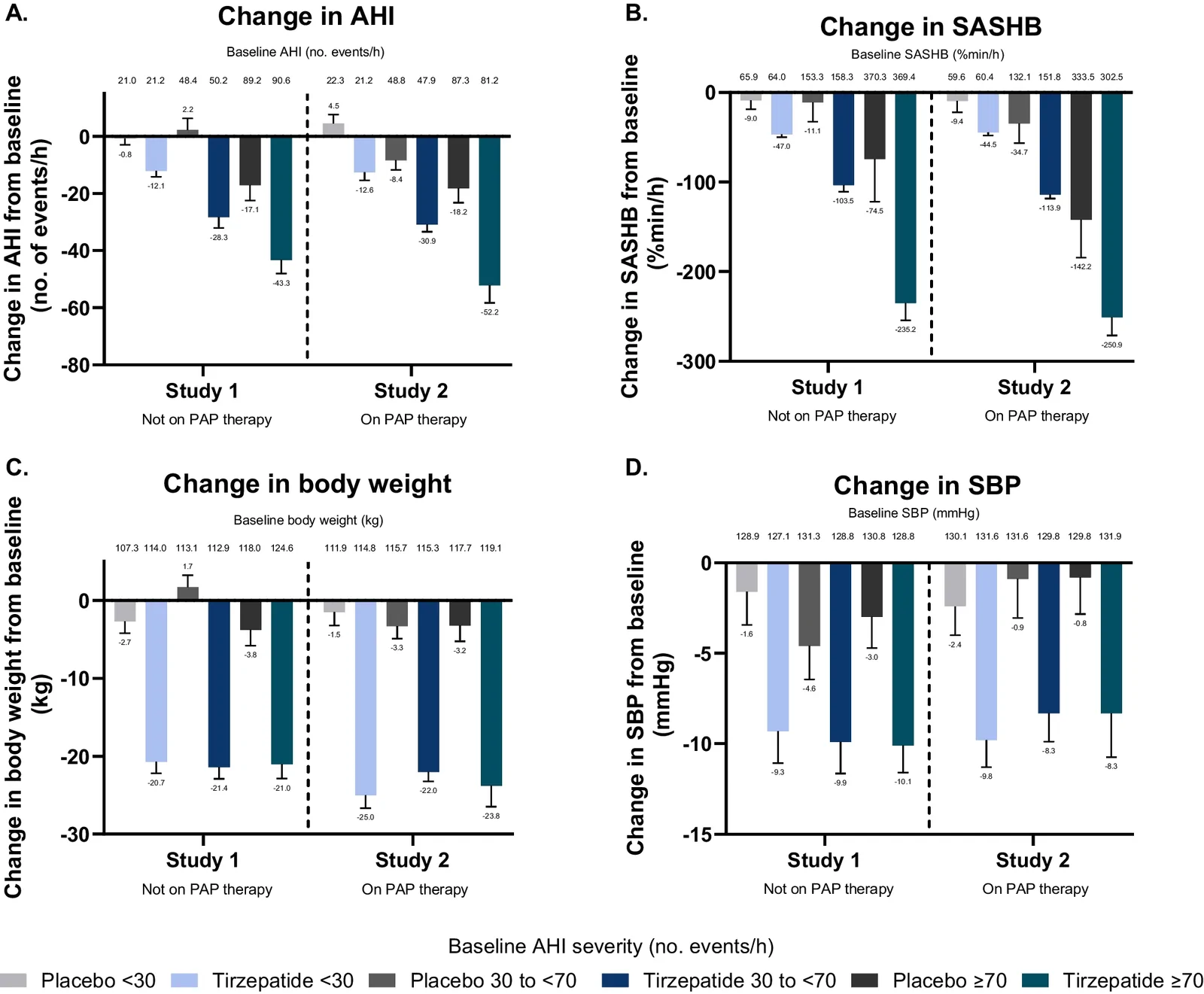
Baseline AHI subgroup. Absolute changes from baseline in participants receiving tirzepatide or placebo in the SURMOUNT-OSA trial by baseline AHI severity in the following measures: **A**. Change in Apnea Hypopnea Index (AHI), **B**. Change in sleep apnea hypoxic burden (SASHB), **C**. Change in body weight (BW), **D**. Change in systolic blood pressure (SBP). Note: Data are model-based estimates with absolute changes listed below the bars and percent changes from baseline listed within or beside the bars, where available

At baseline in both studies, there were more males in the Severe-1 OSA (AHI ≥ 30 to < 70 events/h) and Severe-2 OSA (AHI ≥ 70 events/h) groups, and these groups also tended to have a higher baseline weight, BMI, and SASHB compared with the moderate OSA group (AHI ≥ 15 to < 30 events/h, Table S5). In both studies, tirzepatide treatment was associated with greater reductions in AHI, SASHB, and BW from baseline to Week 52 across all AHI baseline severity groups (Fig. 3A, B, C, Tables S12-13). Tirzepatide-treated participants also had greater reductions in absolute change in SBP across all AHI subgroups and both studies (Fig. 3D, Table S12-13).

For participants treated with tirzepatide, numerically, absolute reductions in AHI were greatest in the Severe-2 OSA subgroup, consistent with higher baseline AHI values in this group. Reductions in the Severe-2 OSA group ranged from −43.3 events/h (Study 1) to −52.2 events/h (Study 2), compared with −12.1 events/h and −12.6 events/h, respectively in the moderate OSA subgroup (Table S12-13). Reductions in BW and SBP were broadly similar across all AHI severity subgroups in both studies.

### BMI

**Fig. 4:**
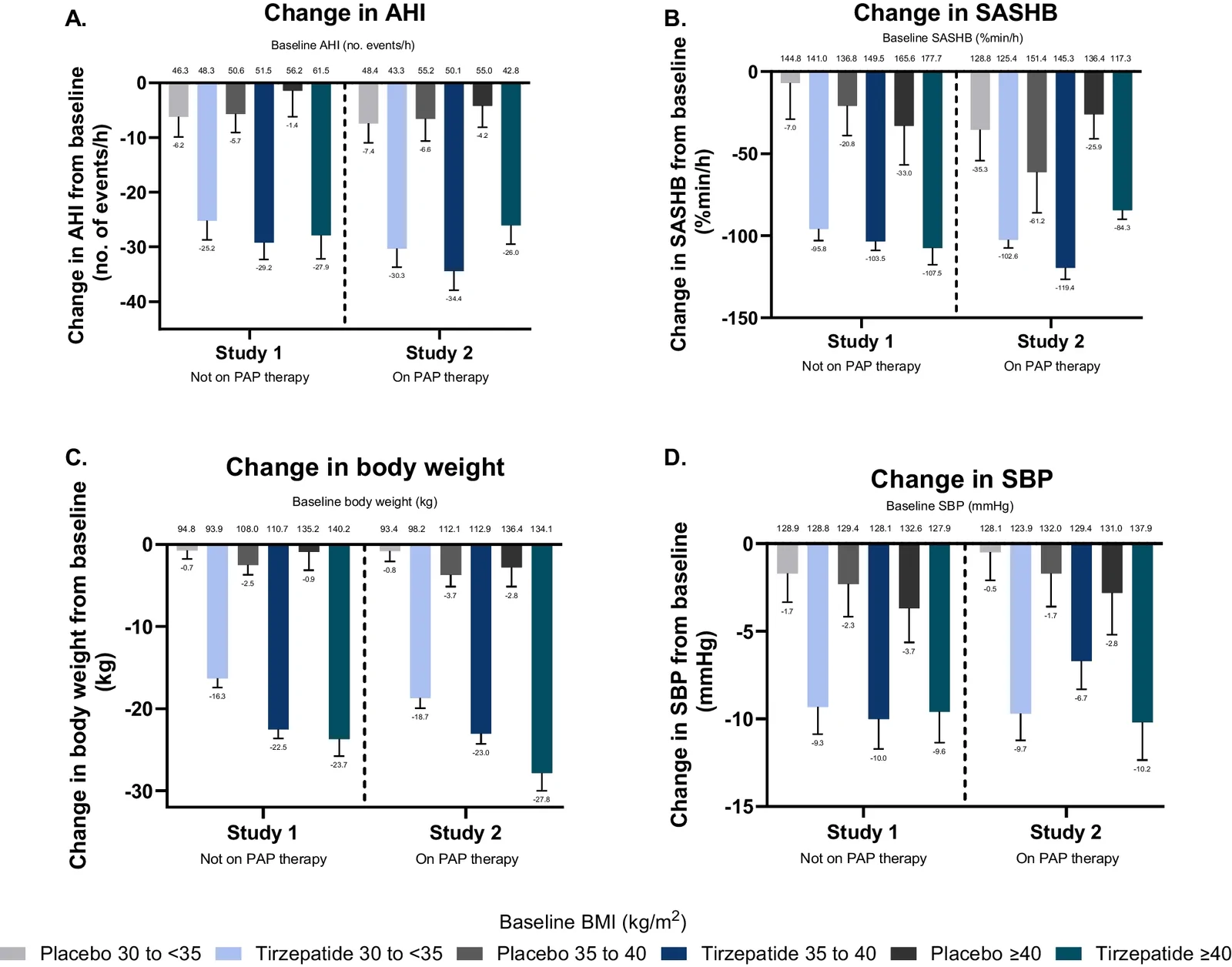
Baseline BMI subgroup. Absolute changes from baseline in participants receiving tirzepatide or placebo in the SURMOUNT-OSA trial by baseline BMI category in the following measures: **A**. Change in Apnea Hypopnea Index (AHI), **B**. Change in sleep apnea hypoxic burden (SASHB), **C**. Change in body weight (BW), **D**. Change in systolic blood pressure (SBP). Note: Data are model-based estimates with absolute changes listed below the bars and percent changes from baseline listed within or beside the bars, where available

Baseline demographics were generally similar across BMI subgroups. However, compared with the lowest BMI subgroup, the highest BMI subgroup was more likely to be younger, female, and have higher BW, BMI, AHI, ESS, SASHB, and SBP (Table S6). Participants treated with tirzepatide experienced a greater reduction in AHI, SASHB, and BW compared with placebo in both studies, across BMI subgroups (Fig. 4A, B, C, Table S14-15). Tirzepatide was also associated with greater absolute reductions in SBP compared with placebo in both studies, regardless of baseline BMI subgroup (Fig. 4D, Table S14-15).

Absolute reductions in AHI were broadly similar across BMI subgroups treated with tirzepatide in both studies (ranging from −25.2 to −34.4 events/h across all subgroups; Table S14-15). Numerically, BW reductions were greater in those with higher baseline BMI, particularly in Study 2 (Table S15).

### Neck circumference (NC)

**Fig. 5:**
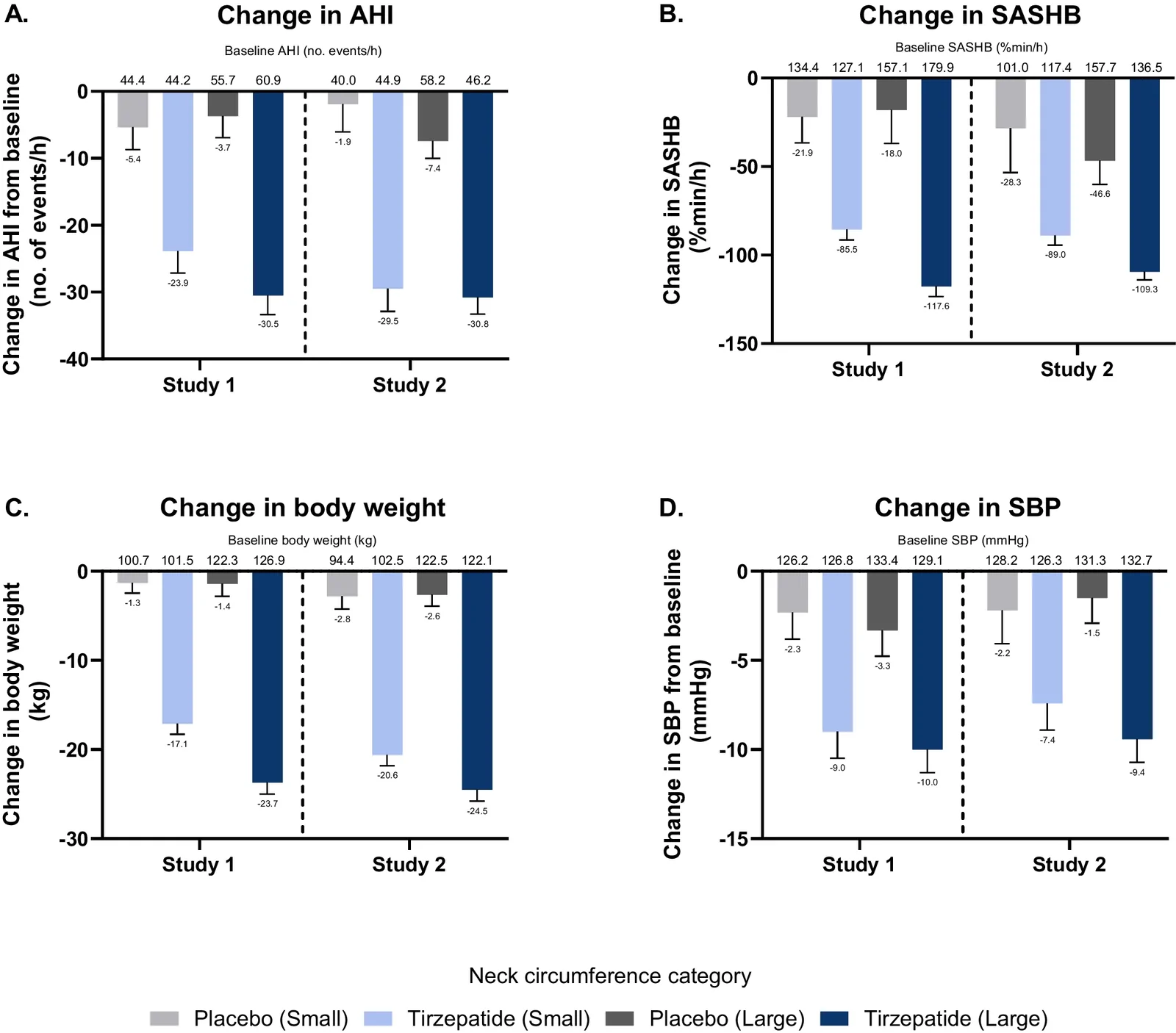
Baseline neck circumference subgroup. Absolute changes from baseline in participants receiving tirzepatide or placebo in the SURMOUNT-OSA trial by baseline neck circumference in the following measures: **A**. Change in Apnea Hypopnea Index (AHI), **B**. Change in sleep apnea hypoxic burden (SASHB), **C**. Change in body weight (BW), **D**. Change in systolic blood pressure (SBP). Note: Data are model-based estimates with absolute changes listed below the bars and percent changes from baseline listed within or beside the bars, where available

In SURMOUNT-OSA, participants with a larger NC tended to be younger, male, and have a higher weight, BMI, AHI, SBP, and SASHB compared with those with smaller neck circumferences (Table S7). Overall, tirzepatide treatment was associated with greater observed improvements in OSA outcomes compared with placebo, in both baseline NC subgroups, in both studies (Fig. 5). A greater reduction in AHI, SASHB, and BW was observed in participants treated with tirzepatide compared with those treated with placebo in both studies, regardless of NC subgroup (Fig. 5A, B, C. Tables S16–17). Additionally, those treated with tirzepatide experienced an improvement in SBP in both studies for each NC subgroup (Fig. 5D, Tables S16–17).

Numerically, absolute reductions in AHI and BW were greater for participants treated with tirzepatide in the large NC subgroup compared with the small NC subgroup, and this pattern was consistent across both studies (AHI: Study 1 large −30.5 events/h versus small −23.9 events/h; Study 2 large −30.8 events/h versus −29.5 events/h; Table S16-S17). SBP reductions were broadly similar across NC subgroups.

### Severity shifts in AHI

**Fig. 6:**
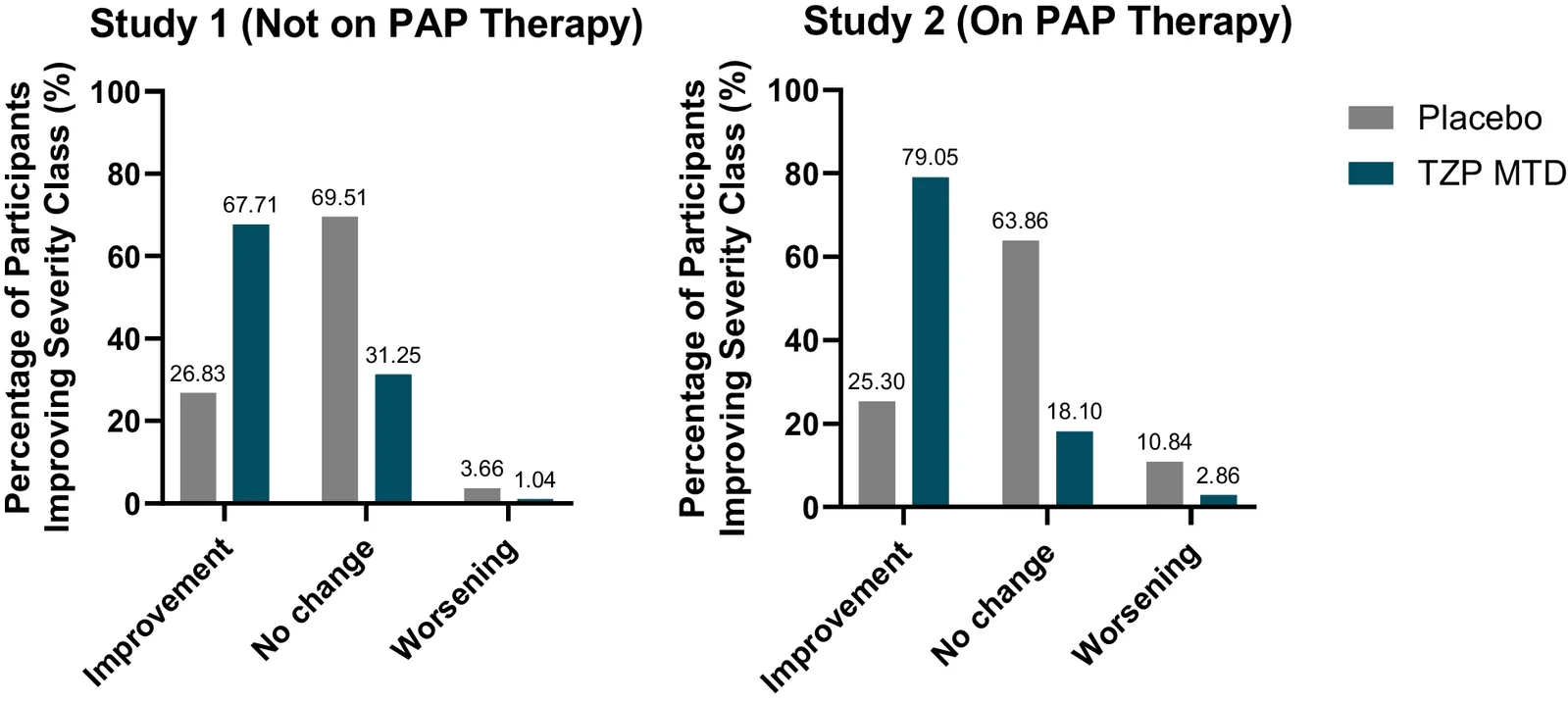
Severity shifts for entire population (improvement, no change and worsening) by placebo and tirzepatide arms. Proportion of participants demonstrating improvement, no change, or worsening in AHI severity category from baseline to Week 52 in the placebo and tirzepatide arms for Study 1 and Study 2. Data are proportions of participants within the efficacy analysis set with non-missing AHI values at baseline and Week 52

In the overall population for Study 1, 67.7% of participants treated with tirzepatide demonstrated an improvement in AHI severity class, while only 26.8% of participants in the placebo arm improved in severity class. In the overall population for Study 2, 79.0% of the tirzepatide group and 25.3% of the placebo group demonstrated an improvement in severity class of AHI (Fig. 6). A greater proportion of participants in the placebo group had either no change [Study 1: 69.5% (placebo), 31.3% (tirzepatide); Study 2: 63.9% (placebo), 18.1% (tirzepatide)] or worsening severity category [Study 1: 3.7% (placebo), 1.0% (tirzepatide); Study 2; 10.8% (placebo), 2.9% (tirzepatide)] (Fig. 6).

Across age subgroups, a higher percentage of participants treated with tirzepatide showed improvement in AHI categories. Regardless of age subgroup, participants in the tirzepatide arm demonstrated more improvements in AHI class (62.4% (< 50 years) and 66.3% (≥ 50 years) in the tirzepatide arm compared with 24.7% (< 50 years) and 25.8% (≥ 50 years) in the placebo arm for Study 1 (Fig. S1, Table S8-9). Study 2 showed more improvements in AHI class for tirzepatide-treated participants (77.3% and 77.8% in the < 50 years and ≥ 50 years subgroups, respectively, and 18.7% and 27.9% for the same subgroups in the placebo arm (Fig. S1-S5, Table S8-9).

The same trends were seen with sex (71.8% of females and 60.5% of males on tirzepatide in Study 1 saw an improvement in AHI class compared with 29.2% of females and 23.1% of males in placebo) (Figure S2, Table S10-11). More severity improvements were also seen in participants treated with tirzepatide in Study 2 (86.1% of females and 74.4% of males on tirzepatide compared with 32.6% of females and 21.0% of males in placebo) (Fig. S2, Table S10-11).

For AHI baseline subgroups, tirzepatide-treated participants experienced more improvements compared with placebo (Figure S3, Table S12-13). The highest percentage demonstrating improvement was seen in the 15—< 30 events/h AHI severity subgroups in both studies (84.8% demonstrated AHI severity class improvement in Study 1 for participants treated with tirzepatide compared with 38.0% of placebo, and in Study 2, 81.4% had improvements in AHI severity class in the treatment arm compared with 30.1% of placebo) (Fig. S3, Table S12-13). Even though the Severe-2 OSA subgroup had the smallest percentage of participants demonstrating improvement in severity classes, those treated with tirzepatide were still more likely to demonstrate improvement than those treated with placebo in both studies (Study 1 – tirzepatide: 31.8%, placebo: 7.6%; Study 2 – tirzepatide: 64.6%, placebo 9.3%).

Regardless of baseline BMI subgroup, participants treated with tirzepatide had a higher percentage of participants demonstrating improvements in AHI severity class compared with those treated with placebo (Fig. S4, Table S14-15). The greatest percentage demonstrating improvement was noted for those in the ≥ 35 to < 40 kg/m^2^ BMI subgroup, with an AHI severity class improvement demonstrated for 70.2% of participants on tirzepatide (32.1% on placebo) in Study 1 and 80.5% of participants on tirzepatide (22.6% on placebo) in Study 2 (Fig. S4, Table S14-15). Those in the highest baseline BMI class exhibited the smallest percentage of participants improving AHI severity classes; however, a higher percentage of those treated with tirzepatide demonstrated severity class improvement, compared with those treated with placebo (Study 1 – tirzepatide: 57.0%, placebo: 20.7%; Study 2 – tirzepatide: 72.7%, placebo 21.0%).

A similar pattern of AHI severity class improvement was seen for those in both baseline NC subgroups, as shown in Figure S5 (71.4% and 60.4% of small and large NC subgroups in the tirzepatide treatment arm compared with 26.7% (small NC) and 24.9% (large NC) in the placebo arm for Study 1). In Study 2, the percent of participants demonstrating improvements in AHI severity class were also observed (80.1% of small NC baseline subgroup and 76.4% of large NC baseline subgroup in the tirzepatide arm compared with 34.8% (small NC) and 20.5% (large NC) of placebo) as shown in Fig. S5, Table S16-17.

## Discussion

In the SURMOUNT-OSA studies, treatment with tirzepatide was associated with improvements in OSA outcomes, regardless of baseline subgroup status across age, sex, AHI severity, BMI, and NC. Although all groups benefited from tirzepatide treatment compared with placebo, some numerical differences in absolute AHI reductions were observed across baseline subgroups. However, as no formal interaction testing was performed between subgroups, these observations are hypothesis-generating and should not be interpreted as confirmed effect modification.

There is an increased risk of OSA with age, independent of weight changes [1, 22]. Age-related increases in OSA are attributable to multiple factors other than BMI change, including co-morbid diseases, reduced upper airway protective reflexes and lung function, and other factors [19]. For these post hoc analyses, in Study 2 (those on PAP therapy), younger participants (< 50 years) achieved numerically greater reductions in AHI and SASHB compared with those in the older cohort (≥ 50 years). Changes in BW were also greater in younger participants in Study 2, consistent with a strong role for obesity in OSA pathogenesis in the younger age group. Participants on PAP therapy in Study 2 by design stopped their PAP therapy for ~ 7 days prior to polysomnography (PSG) measurements. While previous studies have shown a return of OSA during withdrawal, there may be variability in the time course for recurrence of different physiological effects of OSA after PAP withdrawal [20, 21]. Nonetheless, both age subgroups achieved greater improvements in AHI in the tirzepatide-treated arms when compared with placebo. These observations are descriptive in nature, and differences between studies cannot be attributed confidently to PAP withdrawal or any other single factor.

The prevalence of OSA is 2- to threefold higher in males than females, but the prevalence increases in females after menopause [1]. Risk factors for OSA differ for men and women, with women having a generally less collapsible upper airway and a lower arousal threshold than men [18, 22]. In addition to reducing adiposity, tirzepatide may potentially affect multiple OSA pathogenic pathways showing sexual dimorphisms. Numerically, absolute reductions in AHI tended to be greater in males than in females in both studies, consistent with higher baseline AHI in males. This finding did not appear to be explained by larger changes in BW, which was inconsistent across studies. The greater weight reduction observed in females in Study 2 (PAP) is consistent with previous studies with incretin therapies that showed that females tend to lose more weight than males [23, 24]. These differences between studies may reflect the cyclic relationship between untreated OSA and its symptoms and their impact in weight loss, differences in lean versus fat mass between males and females, or possibly differences in pharmacokinetics [24]. For example, both insulin resistance [25] and elevated levels of inflammatory markers [26] have been identified as antecedent risk factors for OSA and may respond more favorably to GLP-1 medications in women than men. While the change in AHI, SASHB, and SBP reflected these BW differences in Study 2, the greater BW change in men in Study 1 was consistent with the SASHB and SBP changes, but not the changes in AHI.

In these post hoc analyses of the SURMOUNT-OSA studies, treatment with tirzepatide was associated with greater improvements in measures of OSA and BW than placebo, irrespective of participants’ OSA severity at baseline or PAP therapy use​. Participants with the highest severity of AHI at baseline (AHI ≥ 70 events/h) tended to have the smallest relative changes in AHI, BW, and SASHB compared with those in the moderate AHI severity subgroup (AHI ≥ 15—< 30 events/h). However, the absolute change in AHI with tirzepatide treatment was greatest in participants with the highest severity of AHI (−43.3 events/h in Study 1 and −52.2 events/h in Study 2), highlighting clinically relevant improvements in this group. In addition, in the highest severity of AHI subgroup (AHI ≥ 70 events/h), over half of participants in both Study 1 (50.6%) and Study 2 (71.1%) had clinically significant reductions in AHI of ≥ 50%.

Increased BMI – as a proxy for adiposity – increases OSA risk through impacts on airway narrowing caused by fat deposition in the upper respiratory tissues [27]. Obesity also contributes to insulin resistance and systemic inflammation [28], each of which may adversely influence muscle function and ventilatory control. These post hoc analyses demonstrated that tirzepatide was associated with improvements in weight reduction and OSA outcomes compared with placebo, regardless of baseline BMI subgroup or use of PAP.

Absolute BW reductions numerically increased with higher baseline BMI, particularly in Study 2, consistent with greater absolute weight reduction expected at higher starting weights. Based on the association between obesity and OSA, it may have been expected that those with higher BW would require additional weight loss to demonstrate improvements in OSA; however, these studies did not support that assertion. In fact, absolute reductions in AHI with tirzepatide treatment compared with placebo appeared broadly similar or even greater in those with the highest BMI (≥ 40 kg/m^2^) compared with those in the lower BMI group (≥ 30 to < 35 kg/m^2^). These results indicate that tirzepatide may be a feasible treatment option for patients with OSA across obesity classes.

Increased NC has been proposed to be a better measure of adiposity-associated risk than BMI in adults with OSA [29, 30]. These post hoc analyses demonstrated that tirzepatide was associated with improvements in weight reduction and OSA outcomes when compared with placebo, regardless of baseline NC subgroup or PAP use. More specific measurements of upper airway fat may identify subgroups with differential treatment responses.

Both obesity and OSA have been associated with increased cardiovascular risk [31]. The intermittent hypoxemia, sleep fragmentation, and large intrathoracic pressure swings are proposed as causal mechanisms of OSA-related cardiometabolic disease [2]. While long-term outcomes were not measured in the SURMOUNT-OSA studies, cardiometabolic risk measures such as insulin resistance, lipids, and BP have been previously reported. Mediation analyses indicate that addressing both OSA and obesity is likely required to optimize treatment effect on cardiometabolic benefits for patients with obesity and moderate-to-severe OSA [10, 32]. In these post hoc analyses, we consistently saw reductions in SBP with tirzepatide treatment in all subgroups in both studies (ranging from 6.7–10.2 mmHg across all subgroups and studies). These are clinically relevant reductions that may improve the overall health of patients with OSA [33]. Traditional OSA therapies such as CPAP have been shown to reduce SBP by 2.4 mmHg at 2 months, with larger improvements reported in patients with excessive sleepiness; however, the magnitude of improvement is not always durable at 6 months [34, 35]. Additional studies to examine the additive effect of PAP therapy and tirzepatide treatment on blood pressure and cardiovascular outcomes are warranted, especially since OSA has been associated with resistant hypertension [36].

Most participants demonstrated improvements in OSA severity class shifts in nearly all baseline subgroups analyzed. The only subgroup that did not have a majority of participants demonstrate improvement was those in the most severe baseline AHI group of ≥ 70 events/h (31.8%) in Study 1. Indeed, a limitation of these analyses was in the categorization used for AHI severity classification. For instance, if a participant falls within the lower range of a category, there may not be much change required to reclassify OSA severity, particularly compared with those with very severe OSA (AHI ≥ 70 events/h). Participants in the higher AHI end have no limit on how many events/h may be recorded at baseline, so would require a larger change in AHI to demonstrate an improvement in AHI severity class. This notion was evident in the baseline AHI subgroup analyses, whereby those in the very severe subgroup (AHI ≥ 70 events/h) have a mean AHI of 91 events/h and 86 events/h in Studies 1 and 2, respectively, compared with 50 and 49 events/h in the AHI ≥ 30 to < 70 events/h subgroup.

While not all participants on tirzepatide reached the criteria to possibly no longer need additional treatments (defined in this study as reaching an AHI < 5 events/h or 5–14 events/h with an ESS ≤ 10), improvements in OSA severity class may open the opportunities for alternative treatment options such as oral appliance treatments or positional therapies that are recommended for those with milder OSA or PAP therapy intolerance.

The current findings should be interpreted in light of the strengths and limitations of these analyses. The SURMOUNT-OSA program included two 52-week, Phase 3, multicenter, parallel-group, double-blind, randomized, placebo-controlled studies across multiple countries, which led to the approval of tirzepatide for moderate-to-severe OSA in adults with obesity in multiple countries. The SURMOUNT-OSA studies were only 52 weeks in duration. Additional weight loss has been demonstrated in studies with longer durations [37–39]; thus, the long-term effects and additional weight reduction on OSA measures are not known. It should be noted that, given the small sample size in these subgroups, the post hoc analyses were not powered for statistical significance. Apparent subgroup differences may reflect chance, residual confounding, differing baseline distributions, or regression to the mean, and additional studies are needed to validate the trends observed here. In addition, aside from the factors controlled for in the models as specified in the statistical analysis section, there may be other potential factors that influence outcomes. Furthermore, other potential confounders not captured in these analyses may influence outcomes, including socioeconomic factors, prior surgical treatments for OSA, PAP adherence, and OSA endotypes and phenotypes. Analyses did not mutually adjust for baseline characteristics, and additional analyses and real-world evidence are needed, using multivariate modeling, to better determine differences and trends across patient populations. Additionally, adverse events and discontinuation of therapy due to side effects, which may have varied across subgroups, were beyond the scope of this study and therefore not reported here. Data after treatment discontinuation were excluded. Discontinuation rates were higher in the placebo arm than the tirzepatide arm across all baseline subgroups. Whether the observed treatment benefit of tirzepatide may have been inflated as a result of this differential drop out pattern warrants further scrutiny. Furthermore, the mechanisms mediating the observed improvements in AHI and SASHB with tirzepatide remain to be fully established. A separate analysis of the SURMOUNT-OSA data demonstrated a linear association between the magnitude of weight reduction and improvements in AHI and SASHB, suggesting that weight loss is an important driver of OSA improvement; however, weight-independent effects of tirzepatide cannot be excluded [40].

## Conclusion

Tirzepatide was associated with improvement in OSA outcomes across two studies, regardless of baseline age, sex, AHI severity, BMI, or NC. While these post hoc studies demonstrated univariate analysis of treatment responses, many pathophysiological mechanisms contribute to OSA, many of which work synergistically to contribute to OSA development. This active research area may help us better understand the influence of baseline characteristics on different OSA treatment responses.

## Supplementary Information

Below is the link to the electronic supplementary material.Supplementary file1 (DOCX 353 KB)

## Data Availability

Eli Lilly and Company provides access to all individual participant data collected during the trial, after anonymization, with the exception of pharmacokinetic or genetic data. Data are available to request 6 months after the indication studied has been approved in the USA and the EU and after primary publication acceptance, whichever is later. No expiration date of data requests is currently set once data are made available. Access is provided after a proposal has been approved by an independent review committee identified for this purpose and after receipt of a signed data sharing agreement. Data and documents, including the study protocol, statistical analysis plan, clinical study report, and blank or annotated case report forms, will be provided in a secure data sharing environment. For details on submitting a request, see the instructions provided at www.vivli.org.
