# Supplementary material for: Association of tirzepatide with changes in OSA-related measures based on baseline characteristics – post hoc analyses of SURMOUNT-OSA
Source: J Clin Sleep Med. 2026 Aug 31;22(1):155. doi: 10.1007/s44470-026-00162-z (PMC13530102; doi:10.1007/s44470-026-00162-z)
Supplement: Supplementary file 1 [file 44470_2026_162_MOESM1_ESM.docx]

**SUPPLEMENTARY FILE**

**Association of tirzepatide with changes in OSA-related measures based on baseline characteristics – Post hoc analyses of SURMOUNT-OSA**

Table of Contents

[SUPPLEMENTARY TABLES	4](#_Toc215760622)

[TABLE S1: Baseline subgroup cut-offs	4](#_Toc215760623)

[**TABLE S2:** Measures in full study data set efficacy estimand receiving treatment	5](#_Toc215760624)

[**TABLE S3:** Baseline and Clinical Characteristics by Age	7](#_Toc215760625)

[**TABLE S4:** Baseline and Clinical Characteristics by Sex	8](#_Toc215760626)

[**TABLE S5:** Baseline and Clinical Characteristics by Baseline AHI subgroups	9](#_Toc215760627)

[**TABLE S6:** Baseline and Clinical Characteristics by baseline BMI subgroups	10](#_Toc215760628)

[**TABLE S7:** Baseline and Clinical Characteristics by baseline Neck Circumference subgroups	11](#_Toc215760629)

[**TABLE S8: Subgroup Age: Study 1:** Change from baseline in participants receiving TZP by SURMOUNT-OSA in multiple measures and targets at Week 52.	12](#_Toc215760630)

[**Table S9: Subgroup Age:** **Study 2:** Change from baseline in participants receiving treatment in SURMOUNT-OSA in multiple measures and targets at Week 52.	14](#_Toc215760631)

[**TABLE S10:** **Subgroup Sex: Study 1:** Change from baseline in participants receiving treatment in SURMOUNT-OSA in multiple measures and targets at week 52.	15](#_Toc215760632)

[**TABLE S11:** **Subgroup Sex: Study 2:** Change from baseline in participants receiving treatment in SURMOUNT-OSA in multiple measures and targets at week 52.	17](#_Toc215760633)

[**TABLE S12:** **Subgroup AHI: Study 1:** Change from baseline in participants receiving treatment in SURMOUNT-OSA in multiple measures and targets at week 52.	19](#_Toc215760634)

[**TABLE S13:** **Subgroup AHI: Study 2:** Change from baseline in participants receiving treatment in SURMOUNT-OSA in multiple measures and targets at week 52.	21](#_Toc215760635)

[**TABLE S14:** **Subgroup BMI: Study 1:** Change from baseline in participants receiving treatment in SURMOUNT-OSA in multiple measures and targets at week 52.	23](#_Toc215760636)

[**TABLE S15:** **Subgroup BMI: Study 2:** Change from baseline in participants receiving treatment in SURMOUNT-OSA in multiple measures and targets at week 52.	25](#_Toc215760637)

[**TABLE S16:** **Subgroup Neck Circumference: Study 1:** Change from baseline in participants receiving treatment in SURMOUNT-OSA in multiple measures and targets at week 52.	27](#_Toc215760638)

[**TABLE S17:** **Subgroup Neck Circumference: Study 2:** Change from baseline in participants receiving treatment in SURMOUNT-OSA in multiple measures and targets at week 52.	29](#_Toc215760639)

[SUPPLEMENTARY FIGURES	32](#_Toc215760640)

[FIGURE S1: % participants with improvement by subgroup Age (PBO and TZP arms)	32](#_Toc215760641)

[FIGURE S2: % participants with improvement by subgroup Sex (PBO and TZP arms)	32](#_Toc215760642)

[FIGURE S3: % participants with improvement by subgroup AHI (PBO and TZP arms)	33](#_Toc215760643)

[FIGURE S4: % participants with improvement by subgroup BMI (PBO and TZP arms)	33](#_Toc215760644)

[FIGURE S5: % participants with improvement by subgroup Neck Circumference (PBO and TZP arms)	34](#_Toc215760645)

# SUPPLEMENTARY TABLES

**Footnote for all tables:**
Data are least-squares means with standard error (SE) or numbers and percent of patients, unless otherwise stated. All changes are from baseline to week 52.

Odds ratios were tirzepatide MTD vs placebo (95% CI)

Differences between TZP and PBO are presented as estimated treatment differences except for the week-52 categories of reduction of ≥50% in AHI events and AHI of <5 or 5 to 14 with ESS ≤10, which are shown as odds ratios. Estimated treatment differences are the differences in the least-squares mean changes.

The least-square mean change from baseline was calculated within each sex group using a mixed model for repeated measurements (MMRM) with Baseline value, Geographic Region, Baseline OSA Severity Group, Sex, Treatment, Time, and Treatment*Time as the fixed effect and patient as random effect. A full MMRM model with sex and interaction terms provided the p-value for comparing treatment effects by sex at specific time intervals. Odds ratios were calculated from a logistic regression with baseline value, geographic region, and treatment as covariates, where missing data at week 52 were imputed using multiple imputation in each treatment arm, assuming missing at random.

Efficacy analyses were conducted in all randomized participants who received ≥1 treatment dose. Data obtained after discontinuation of study intervention or initiation of PAP therapy (study 1 only) were excluded from analysis.

Abbreviations: AHI, apnea–hypopnea index; BMI, body mass index; PBO, placebo; SASHB, sleep apnea–specific hypoxic burden; SE, standard error; TZP, tirzepatide.

## **TABLE S1:** Baseline subgroup cut-offs

| Subgroup | Cut-off | Study 1 (N) | Study 2 (N) |
| --- | --- | --- | --- |
| Age* | <50 years | 125 | 98 |
|  | ≥50 years | 109 | 135 |
| Sex | Male | 157 | 169 |
|  | Female | 77 | 64 |
| AHI† | ≥15/h to <30/h (Moderate OSA) | 86 | 74 |
|  | ≥30/h to <70/h  (Severe-1 OSA*) | 77 | 99 |
|  | ≥70/h  (Severe-2 OSA*) | 70 | 60 |
| BMI‡ | ≥30 to < 35 kg/m^2^  (Class I obesity) | 77 | 66 |
|  | ≥35 to < 40 kg/m^2^  (Class II obesity) | 74 | 88 |
|  | ≥ 40 kg/m^2^  (Class III obesity) | 83 | 79 |
| Neck circumference (NC)§ | <41 cm (females)  <43 cm (males)  (Small NC) | 95 | 68 |
|  | ≥41 cm (females)  ≥43 cm (males)  Large NC | 136 | 165 |

*Age cut-off was predefined in the study protocol

†AHI cut-offs were determined by OSA severity classification. Severe OSA was further divided into Severe-1 and Severe-2 subgroups to establish approximately evenly weighted subgroups[1]. Baseline mean values and ranges for combined tirzepatide and placebo treatment arms by subgroup are as follows. Study 1: Moderate 20.6 events/h (3.2 to 29.9 event/h), Severe-1 50.0 events/h (30.5 to 69.3 events/h), Severe-2 91.1 events/h (72.2 to 146.9 events/h); Study 2: Moderate 21.2 events/h (6.6 to 29.9 events/h), Severe-1 48.6 events/h (30.5 to 69.6 events/h), Severe-2 85.9 events/h (71.0 to 120.7 events/h) (means found in Table S5).

‡BMI cut-offs were determined by obesity class.

§NC subgroups were based on risk factor cut-offs used in the STOP-BANG screening tool [1].

## **TABLE S2:** Measures in full study data set efficacy estimand receiving treatment

| **OSA Outcome** | **Study 1 – Not on PAP Therapy** | | | **Study 2 – On PAP Therapy** | | |
| --- | --- | --- | --- | --- | --- | --- |
|  | **PBO** | **TZP** | **Treatment difference** | **PBO** | **TZP** | **Treatment difference** |
| Change in AHI (SE), no. of events/h | -4.8 (2.28) | -27.4 (2.14) | -22.5 (-28.7, -16.4)*** | -6.0 (2.19) | -30.4 (1.98) | -24.4 (-30.3, -18.6)*** |
| % Change AHI (SE) | -3.0 (7.07) | -50.7 (5.90) | -47.7 (-65.76, -29.55)*** | -2.5 (6.95) | -58.7 (5.28) | -56.2 (-73.73, -38.70)*** |
| Change in SASHB (SE), %min/h | -21.1 (12.02) | -103.1 (4.21) | -82.0 (12.73) | -40.7 (12.11) | -103.0 (3.63) | -62.4 (12.65) |
| % change SASHB | -13.8 (7.88) | -67.6 (2.76) | -62.4 (-70.6, -51.9)*** | -30.4 (9.04) | -76.9 (2.71) | -66.8 (-76.5, -53.1)*** |
| Change Body Weight, kg | -1.3 (0.96) | -21.0 (0.93) | -19.8 (-22.4, -17.1)*** | -2.6 (1.02) | -23.3 (0.96) | -20.6 (-23.4, -17.9)*** |
| % Change BW | -1.3 (0.73) | -18.1 (0.71) | -16.8 (-18.8, -14.7)*** | -2.3 (0.78) | -20.1 (0.73) | -17.8 (-19.9, -15.7)*** |
| Change SBP, mmHg | -2.9 (1.04) | -9.7 (0.97) | -6.8 (-9.6, -4.0)*** | -1.6 (1.13) | -8.7 (1.01) | -7.2 (-10.2, -4.2)*** |
| OR achieving ≥50% AHI reduction | 19.2 (4.31) | 62.3 (4.88) | 7.53 (3.75, 15.15)*** | 22.9 (4.45) | 74.3 (4.21) | 9.20 (4.75, 17.82)*** |
| OR achieving AHI <5 or 5-14 with ESS ≤10 | 14.9 (3.89) | 43.0 (4.89) | 8.96 (3.55, 22.61)*** | 13.6 (3.69) | 51.5 (4.77) | 8.12 (3.60, 18.33)*** |
| Change in hsCRP (mg/L) | -0.79 (0.24) | -1.64 (0.16) | -0.84 (-1.42, -0.27) | -0.29 (0.28) | -1.44 (0.14) | -1.14 (-1.75, -0.54) |
| % Change hsCRP (mg/L) | -21.4 (6.59) | -44.2 (4.40) | -28.9 (-43.4, - 10.8)** | -10.4 (9.78) | -50.7 (4.78) | -45.1 (-58.8, -26.7)*** |

Note: Data are LSMean (SE), MiTT population, Efficacy Analysis Set. Treatment difference = Placebo adjusted. OR = Odds Ratio. * p < 0.05; ** p < 0.01; *** p < 0.001. PAP = Positive Airway Pressure; F= Female; BMI = body mass index; AHI = apnea-hypoapnea index; ESS = Epworth sleepiness scale; SASHB = sleep apnea-specific hypoxic burden (total hypoxic burden); hsCRP = high sensitivity C-reactive protein

## **TABLE S3:** Baseline and Clinical Characteristics by Age

| **Baseline characteristic** | **Study 1 – Not on PAP Therapy** | | **Study 2 – On PAP Therapy** | |
| --- | --- | --- | --- | --- |
|  | **<50 yrs**  **N=125** | **≥50 yrs**  **N=109** | **<50 yrs N=98** | **≥50 yrs**  **N=135** |
| Age, yrs, Mean (SD) | 39.4 (7.2) | 57.6 (6.7) | 41.2 (5.4) | 59.3 (7.1) |
| Sex, F, n (%) | 30 (24.0) | 47 (43.1) | 15 (15.3) | 49 (36.3) |
| Weight, kg, Mean (SD) | 120.59 (23.23) | 107.88 (22.37) | 121.94 (20.74) | 110.76 (21.82) |
| BMI, kg/m^2^, Mean (SD) | 40.46 (7.39) | 37.54 (6.25) | 39.61 (6.49) | 37.96 (5.55) |
| AHI, no. events/h, Mean (SD) | 55.03 (33.07) | 47.41 (27.99) | 55.66 (28.61) | 45.05 (24.32) |
| ESS, Mean (SD) | 10.44 (5.27) | 10.75 (5.26) | 10.58 (4.55) | 9.89 (4.50) |
| SASHB, % min/h, Mean (SD) | 236.23 (215.27) | 176.81 (148.67) | 210.87 (183.04) | 180.00 (167.73) |
| SBP, mmHg, Mean (SD) | 129.7 (10.58) | 128.8 (12.30) | 129.4 (13.18) | 131.4 (13.75) |

Note: Data are LSMean (SD), MiTT population, Efficacy Analysis Set. PAP = Positive Airway Pressure; F= Female; BMI = body mass index; AHI = apnea-hypoapnea index; ESS = Epworth sleepiness scale; SASHB = sleep apnea-specific hypoxic burden (total hypoxic burden)

## **TABLE S4:** Baseline and Clinical Characteristics by Sex

| **Baseline characteristic** | **Study 1 – Not on PAP Therapy** | | **Study 2 – On PAP Therapy** | |
| --- | --- | --- | --- | --- |
|  | **Female**  **N=77** | **Male**  **N=157** | **Female**  **N=64** | **Male**  **N=169** |
| Age, yrs, Mean (SD) | 51 (9.4) | 46.2 (12.0) | 56.6 (10.0) | 49.8 (10.8) |
| Weight, kg, Mean (SD) | 107.79 (24.67) | 118.04 (22.46) | 105.67 (22.47) | 119.17 (20.75) |
| BMI, kg/m^2^, Mean (SD) | 40.97 (8.50) | 38.18 (5.99) | 39.96 (7.34) | 38.16 (5.35) |
| AHI, no. events/h, Mean (SD) | 39.42 (28.54) | 57.41 (30.47) | 35.80 (20.49) | 54.71 (26.95) |
| ESS, Mean (SD) | 10.58 (5.57) | 10.59 (5.11) | 10.02 (3.97) | 10.26 (4.73) |
| SASHB, % min/h, Mean (SD) | 133.07 (125.08) | 245.63 (203.98) | 106.14 (104.38) | 225.87 (184.55) |
| SBP, mmHg, Mean (SD) | 127.4 (13.35) | 130.2 (10.16) | 129.1 (15.65) | 131.2 (12.62) |

Note: Data are LSMean (SD), MiTT population, Efficacy Analysis Set. PAP = Positive Airway Pressure; F= Female; BMI = body mass index; AHI = apnea-hypoapnea index; ESS = Epworth sleepiness scale; SASHB = sleep apnea-specific hypoxic burden (total hypoxic burden)

## **TABLE S5:** Baseline and Clinical Characteristics by Baseline AHI subgroups

| **Baseline characteristic** | **Study 1 – Not on PAP Therapy** | | | **Study 2 – On PAP Therapy** | | |
| --- | --- | --- | --- | --- | --- | --- |
|  | **Moderate**  **15 - <30**  **N=86** | **Severe 1**  **≥30 - <70**  **N=77** | **Severe 2**  **≥70**  **N=70** | **Moderate**  **15 - <30**  **N=74** | **Severe 1**  **≥30 - <70**  **N=99** | **Severe 2**  **≥70**  **N=60** |
| Age, yrs, Mean (SD) | 48.6 (12.0) | 50.6 (11.1) | 44.5 (10.0) | 54.4 (10.8) | 51.4 (10.9) | 48.9 (10.8) |
| Sex, F, n (%) | 43 (50.0) | 24 (31.2) | 10 (14.3) | 34 (45.9) | 23 (23.2) | 7 (11.7) |
| Weight, kg, Mean (SD) | 110.26 (19.87) | 112.91 (25.54) | 121.38 (24.13) | 113.27 (22.86) | 115.45 (22.03) | 118.19 (21.03) |
| BMI, kg/m^2^, Mean (SD) | 38.53 (6.90) | 38.56 (7.00) | 40.34 (7.17) | 38.29 (6.06) | 38.71 (6.30) | 39.01 (5.47) |
| AHI, no. events/h, Mean (SD) | 20.61 (5.14) | 49.95 (11.81) | 91.05 (16.10) | 21.20 (5.08) | 48.64 (12.70) | 85.88 (12.09) |
| ESS, Mean (SD) | 10.07 (5.19) | 9.75 (5.07) | 12.15 (5.33) | 10.24 (4.97) | 10.11 (4.19) | 10.25 (4.53) |
| SASHB, % min/h, Mean (SD) | 68.50 (28.38) | 189.01 (141.81) | 401.71 (185.21) | 63.28 (20.87) | 173.07 (113.41) | 385.81 (196.00) |
| SBP, mmHg, Mean (SD) | 128.1 (11.47) | 130.0 (11.42) | 129.8 (11.35) | 130.8 (13.21) | 130.4 (14.35) | 130.5 (12.67) |

Note: Data are LSMean (SD), MiTT population, Efficacy Analysis Set. PAP = Positive Airway Pressure; F= Female; BMI = body mass index; AHI = apnea-hypoapnea index; ESS = Epworth sleepiness scale; SASHB = sleep apnea-specific hypoxic burden (total hypoxic burden)

## **TABLE S6:** Baseline and Clinical Characteristics by baseline BMI subgroups

| **Baseline characteristic** | **Study 1 – Not on PAP Therapy** | | | **Study 2 – On PAP Therapy** | | |
| --- | --- | --- | --- | --- | --- | --- |
|  | **30 - <35**  **N=77** | **≥35 - <40**  **N=74** | **≥40**  **N=83** | **30 - <35**  **N=66** | **≥35 - <40**  **N=88** | **≥40**  **N=79** |
| Age, yrs, Mean (SD) | 50.0 (11.8) | 48.5 (12.4) | 45.3 (9.8) | 52.2 (10.4) | 53.3 (11.1) | 49.5 (11.2) |
| Sex, F, n (%) | 25 (32.5) | 18 (24.3) | 34 (41.0) | 16 (24.2) | 20 (22.7) | 28 (35.4) |
| Weight, kg, Mean (SD) | 94.97 (11.86) | 109.29 (12.87) | 137.75 (19.56) | 95.84 (11.68) | 112.57 (12.60) | 135.08 (20.68) |
| BMI, kg/m^2^, Mean (SD) | 32.74 (1.60) | 37.02 (1.36) | 48.86 (5.76) | 32.49 (1.64) | 37.33 (1.38) | 45.27 (4.93) |
| AHI, no. events/h, Mean (SD) | 44.43 (24.32) | 49.87 (29.76) | 59.52 (35.76) | 46.02 (24.67) | 51.51 (26.48) | 50.22 (28.49) |
| ESS, Mean (SD) | 9.82 (5.01) | 10.97 (5.58) | 10.95 (5.17) | 9.26 (4.43) | 10.44 (4.57) | 10.73 (4.49) |
| SASHB, % min/h, Mean (SD) | 185.92 (179.14) | 202.67 (187.24) | 234.77 (198.60) | 180.58 (161.83) | 207.71 (186.05) | 186.95 (172.75) |
| SBP, mmHg, Mean (SD) | 128.8 (12.26) | 128.7 (9.29) | 130.2 (12.31) | 126.0 (11.49) | 130.6 (13.79) | 134.4 (13.76) |

Note: Data are LSMean (SD), MiTT population, Efficacy Analysis Set. PAP = Positive Airway Pressure; F= Female; BMI = body mass index; AHI = apnea-hypoapnea index; ESS = Epworth sleepiness scale; SASHB = sleep apnea-specific hypoxic burden (total hypoxic burden)

## **TABLE S7:** Baseline and Clinical Characteristics by baseline Neck Circumference subgroups

| **Baseline characteristic** | **Study 1 – Not on PAP Therapy** | | **Study 2 – On PAP Therapy** | |
| --- | --- | --- | --- | --- |
|  | **Small NC**  **N=95** | **Large NC**  **N=136** | **Small NC**  **N=68** | **Large NC**  **N=165** |
| Age, yrs, Mean (SD) | 48.7 (11.3) | 47.3 (11.7) | 54.0 (11.1) | 50.7 (10.9) |
| Sex, F, n (%) | 44 (46.3) | 33 (24.3) | 34 (50.0) | 30 (18.2) |
| Weight, kg, Mean (SD) | 101.09 (17.94) | 124.20 (22.75) | 98.95 (13.87) | 122.27 (21.17) |
| BMI, kg/m^2^, Mean (SD) | 36.03 (5.78) | 41.27 (7.06) | 35.01 (3.75) | 40.15 (6.12) |
| AHI, no. events/h, Mean (SD) | 43.64 (25.67) | 56.82 (33.19) | 42.14 (23.83) | 52.56 (27.25) |
| ESS, Mean (SD) | 10.91 (5.66) | 10.26 (4.92) | 9.00 (4.29) | 10.69 (4.53) |
| SASHB, % min/h, Mean (SD) | 177.73 (161.31) | 227.84 (203.55) | 161.53 (171.99) | 205.94 (174.55) |
| SBP, mmHg, Mean (SD) | 126.5 (11.30 ) | 131.2 (11.02) | 127.1 (14.12) | 132.0 (13.05) |

Note: Data are LSMean (SD), MiTT population, Efficacy Analysis Set. PAP = Positive Airway Pressure; F= Female; BMI = body mass index; AHI = apnea-hypoapnea index; ESS = Epworth sleepiness scale; SASHB = sleep apnea-specific hypoxic burden (total hypoxic burden). Note 2: Baseline Neck Circumference size: Small: <41cm (females); <43cm (males); Large: ≥41cm (females); ≥43cm (males)

## **TABLE S8: Subgroup Age: Study 1:** Change from baseline in participants receiving TZP by SURMOUNT-OSA in multiple measures and targets at Week 52.

|  | **Study 1 – Not on PAP Therapy** | | | | | |
| --- | --- | --- | --- | --- | --- | --- |
|  | **<50 yrs** | | | **≥50 yrs** | | |
| **OSA Outcome** | **PBO** | **TZP** | **Treatment difference** | **PBO** | **TZP** | **Treatment difference** |
| Change in AHI (SE), no. of events/h | -4.9 (3.54) | -27.9 (3.01) | -22.9 (-32.2, -13.7) *** | -4.0 (2.94) | -27.8 (3.08) | -23.7 (-32.3, -15.2) *** |
| % Change AHI (SE) | -7.0 (7.09) | -55.0 (6.00) | -48.0 (-66.5, -29.4) *** | -2.5 (6.7) | -55.8 (6.99) | -53.2 (-72.7, -33.8) *** |
| Change in SASHB (SE), %min/h | -27.6 (19.66) | -113.2 (6.37) | -85.5 (-126.0, -45.0) | -14.1 (14.81) | -92.9 (5.57) | -78.8 (-109.8, -47.8) |
| % change SASHB | -16.6 (11.82) | -68.1 (3.83) | -61.7 (-73.6, -44.5) *** | -10.2. (10.75) | -67.4 (4.05) | -63.7(-74.3, -48.7) *** |
| Change Body Weight, kg | -0.7 (1.43) | -21.2 (1.27) | -20.5 (-24.3, -16.7)*** | -2.0 (1.32) | -20.7 (1.39) | -18.7 (-22.5, -14.9)*** |
| % Change BW | -0.7 (1.09) | -17.6 (0.97) | -16.8 (-19.7, -13.9)*** | -2.0 (0.99) | -18.8 (1.04) | -16.8 (-19.6, -13.9)*** |
| Change SBP, mmHg | -3.6 (1.48) | -9.9 (1.26) | -6.4 (-10.2, -2.5) ** | -2.3 (1.49) | -9.1 (1.56) | -6.8 (-11.1, -2.4) ** |
| OR achieving ≥50% AHI reduction | 22.5 (6.17) | 59.38 (6.55) | 4.93 (2.01, 12.10) *** | 15.7 (5.50) | 65.9 (7.34) | 11.25 (3.75, 33.74) *** |
| OR achieving AHI <5 or 5-14 with ESS ≤10 | 14.2 (5.20) | 47.4 (6.53) | 9.8 (2.71, 35.41)*** | 15.7 (5.32) | 37.7 (7.35) | 9.1 (2.16, 38.05)** |
| Severity Shifts | 24.7 (6.36) | 62.4 (6.41) | 7.9 (2.64, 23.71)*** | 25.8 (6.27) | 66.3 (7.27) | 8.6 (2.82, 26.43)*** |

Note: Data are LSMean (SE), MiTT population, Efficacy Analysis Set. OR = Odds Ratio. * p < 0.05; ** p < 0.01; *** p < 0.001. P-values were not calculated for change in SASHB treatment difference.

Note 2: “Treatment Difference” column refers to Odds Ratio (95% CI), where CI = Confidence interval.

## **Table S9: Subgroup Age:** **Study 2:** Change from baseline in participants receiving treatment in SURMOUNT-OSA in multiple measures and targets at Week 52.

| **OSA Outcome** | **Study 2 – On PAP Therapy** | | | | | |
| --- | --- | --- | --- | --- | --- | --- |
|  | **<50 yrs** | | | **≥50 yrs** | | |
|  | **PBO** | **TZP** | **Treatment difference** | **PBO** | **TZP** | **Treatment difference** |
| Change in AHI (SE), no. of events/h | -5.6 (3.86) | -34.1 (3.14) | -28.6 (-38.6, -18.5)*** | -5.8 (2.68) | -27.7 (2.58) | -21.9 (-29.3, -14.5)*** |
| % Change AHI (SE) | -0.2 (9.99) | -66.4 (8.16) | -66.2 (-92.1, -40.3) *** | -11.1 (6.04) | -59.0 (5.74) | -47.9 (-64.5, -31.3) *** |
| Change in SASHB (SE), %min/h | -37.7 (24.25) | -114.1 (5.16) | -76.4 (-125.0, -27.8) | -42.7 (12.98) | -93.6 (5.16) | -50.8 (-78.2, -23.5) |
| % change SASHB | -26.7 (17.22) | -81.0 (3.67) | -74.1 (-85.9, -52.5) *** | -33.0 (10.03) | -72.2 (3.99) | -58.6 (-72.6, -37.5) *** |
| Change Body Weight, kg | -1.5 (1.69) | -24.4 (1.47) | -22.9 (-27.4, -18.5)*** | -3.5 (1.28) | -22.2 (1.27) | -18.7 (-22.3, -15.1)*** |
| % Change BW | -1.3 (1.28) | -20.3 (1.11) | -19.0 (-22.3, -15.6)*** | -3.1 (1.00) | -19.9 (0.98) | -16.8 (-19.6, -14.0)*** |
| Change SBP, mmHg | -1.7 (1.77) | -7.8 (1.45) | -6.1 (-10.7, -1.6) ** | -1.4 (1.48) | -9.5 (1.41) | -8.1 (-12.2, -4.1) *** |
| OR achieving ≥50% AHI reduction | 19.6 (6.67) | 77.5 (6.07) | 12.17 (4.06, 36.52)*** | 25.1 (5.80) | 71.8 (5.83) | 7.02 (3.07, 16.04)*** |
| OR achieving AHI <5 or 5-14 with ESS ≤10 | 9.9 (5.06) | 55.4 (7.02) | 14.2 (3.19, 62.86)*** | 16.0 (5.04) | 48.4 (6.47) | 5.7 (2.14, 15.20)*** |
| Severity Shifts | 18.7 (6.60) | 77.3 (6.04) | 13.2 (4.10, 42.25)*** | 27.9 (5.94) | 77.8 (5.37) | 9.3 (3.85, 22.46)*** |

Note: Data are LSMean (SE), MiTT population, Efficacy Analysis Set. OR = Odds Ratio. * p < 0.05;** p < 0.01;*** p < 0.001. P-values were not calculated for change in SASHB treatment difference.

Note 2: “Treatment Difference” column refers to Odds Ratio (95% CI), where CI = Confidence interval.

## **TABLE S10:** **Subgroup Sex: Study 1:** Change from baseline in participants receiving treatment in SURMOUNT-OSA in multiple measures and targets at week 52.

| **OSA Outcome** | **Study 1 – Not on PAP Therapy** | | | | | |  |
| --- | --- | --- | --- | --- | --- | --- | --- |
|  | **Female** | | | **Male** | | | |
|  | **PBO** | **TZP** | **Treatment difference** | **PBO** | **TZP** | **Treatment difference** | |
| Change in AHI (SE), no. of events/h | -1.1 (3.90) | -19.8 (3.86) | -18.8 (-29.9, -7.6)** | -6.6 (2.85) | -30.6 (2.59) | -23.9 (-31.6, -16.3)*** | |
| % Change AHI (SE) | -3.8 (10.32) | -51.5 (10.18) | -55.3 (-84.6, -25.9)*** | -9.5 (5.16) | -56.2 (4.68) | -46.7 (-60.5, -32.9)*** | |
| Change in SASHB (SE), %min/h | -12.8 (14.20) | -67.1 (5.78) | -54.3 (-84.4, -24.3) | -25.8 (18.09) | -127.1 (5.84) | -101.3 (-138.6, -64.1) | |
| % change SASHB | -12.1 (13.45) | -63.6 (5.47) | -58.6 (-73.1, -36.2)*** | -14.1 (9.89) | -69.5 (3.19) | -64.5 (-73.9, -51.7)*** | |
| Change Body Weight, kg | -1.7 (1.58) | -17.2 (1.60) | -15.5 (-20.0, -11.0)*** | -1.1 (1.18) | -22.9 (1.11) | -21.8 (-25.0, -18.6)*** | |
| % Change BW | -1.6 (1.37) | -16.3 (1.39) | -14.6 (-18.5, -10.7)*** | -1.2 (0.85) | -19.0 (0.80) | -17.7 (-20.1, -15.4)*** | |
| Change SBP, mmHg | -3.2 (2.00) | -9.8 (1.95) | -6.6 (-12.1, -1.0)* | -2.6 (1.2) | -9.5 (1.10) | -6.9 (-10.1, -3.6)*** | |
| OR achieving ≥50% AHI reduction | 19.8 (7.54) | 68.1 (8.53) | 11.0 (2.92, 41.78)*** | 18.9 (5.06) | 59.6 (5.96) | 6.3 (2.78, 14.09)*** | |
| OR achieving AHI <5 or 5-14 with ESS ≤10 | 22.9 (7.86) | 51.9 (9.02) | 9.3 (2.08, 41.61)** | 10.8 (4.04) | 39.0 (5.81) | 8.5 (2.70, 26.65)*** | |
| Severity Shifts | 29.2 (8.39) | 71.8 (8.15) | 13.3 (2.92, 60.20)*** | 23.1 (5.31) | 60.5 (5.90) | 6.9 (2.81, 17.13)*** | |

Note: Data are LSMean (SE), MiTT population, Efficacy Analysis Set. PAP = Positive Airway Pressure; AHI = apnea-hypoapnea index; SASHB = sleep apnea-specific hypoxic burden (total hypoxic burden); BW = Body Weight; SBP = Systolic Blood Pressure; OR = Odds Ratio. * p < 0.05; ** p < 0.01; *** p < 0.001

Note 2: “Treatment Difference” column refers to Odds Ratio (95% CI), where CI = Confidence interval.

## **TABLE S11:** **Subgroup Sex: Study 2:** Change from baseline in participants receiving treatment in SURMOUNT-OSA in multiple measures and targets at week 52.

| **OSA Outcome** | **Study 2 – On PAP Therapy** | | | | | |
| --- | --- | --- | --- | --- | --- | --- |
|  | **Female** | | | **Male** | | |
|  | **PBO** | **TZP** | **Treatment difference** | **PBO** | **TZP** | **Treatment difference** |
| Change in AHI (SE), no. of events/h | -1.8 (3.49) | -24.8 (3.24) | -23.1 (-32.6, -13.5)*** | -7.5 (2.78) | -32.6 (2.48) | -25.1 (32.6, -17.7)*** |
| % Change AHI (SE) | -2.0 (11.33) | -66.8 (10.51) | -64.8 (-95.8, -33.7)*** | -8.2 (6.07) | -61.2 (5.39) | -52.9 (-69.1, -36.8)*** |
| Change in SASHB (SE), %min/h | -16.8 (13.62) | -65.1 (3.74) | -48.2 (-75.9, -20.5) | -55.7 (16.20) | -122.6 (5.20) | -67.0 (-100.3, -33.6) |
| % change SASHB | -19.8 (16.01) | -76.5 (4.39) | -70.7 (-83.1, -49.2)*** | -34.7 (10.10) | -76.5 (3.25) | -64.0 (-76.1, -45.7)*** |
| Change Body Weight, kg | -1.3 (1.50) | -24.8 (1.44) | -23.5 (-27.7, -19.4) | -3.1 (1.29) | -22.7 (1.2) | -19.6 (-23.0, -16.1) |
| % Change BW | -1.7 (1.29) | -23.1 (1.23) | -21.4 (-25.0, -17.9)*** | -2.5 (0.95) | -18.9 (0.87) | -16.4 (-18.9, -13.8)*** |
| Change SBP, mmHg | 0.6 (2.20) | -9.2 (2.02) | -9.7 (-15.7, -3.7)** | -2.7 (1.30) | -8.6 (1.14) | -5.9 (-9.3, -2.5)*** |
| OR achieving ≥50% AHI reduction | 25.8 (8.33) | 75.2 (8.04) | 7.8 (2.32, 26.52)*** | 21.8 (5.22) | 74.0 (4.96) | 9.5 (4.34, 20.78)*** |
| OR achieving AHI <5 or 5-14 with ESS ≤10 | 19.2 (7.49) | 68.9 (8.65) | 9.2 (2.41, 35.20)** | 11.4 (4.14) | 45.1 (5.52) | 7.1 (2.64, 19.06)*** |
| Severity Shifts | 32.6 (8.82) | 86.1 (6.39) | 12.0 (3.05, 47.09)*** | 21.0 (5.09) | 74.4 (4.91) | 10.2 (4.60, 22.71)*** |

Note: Data are LSMean (SE), MiTT population, Efficacy Analysis Set. OR = Odds Ratio. * p < 0.05; ** p < 0.01; *** p < 0.001

Note 2: “Treatment Difference” column refers to Odds Ratio (95% CI), where CI = Confidence interval.

## **TABLE S12:** **Subgroup AHI: Study 1:** Change from baseline in participants receiving treatment in SURMOUNT-OSA in multiple measures and targets at week 52.

| **OSA Outcome** | **Study 1 – Not on PAP Therapy** | | | | | | | | |
| --- | --- | --- | --- | --- | --- | --- | --- | --- | --- |
|  | **15 - <30** | | | **30 - <70** | | | **≥70** | | |
|  | **PBO** | **TZP** | **Treatment difference** | **PBO** | **TZP** | **Treatment difference** | **PBO** | **TZP** | **Treatment difference** |
| Change AHI, no. of events/h | -0.8 (2.23) | -12.1 (2.11) | -11.3 (-17.4, -5.2)*** | 2.2 (4.04) | -28.3 (3.88) | -30.5 (-41.8, -19.3)*** | -17.1 (5.36) | -43.3 (4.74) | -26.2 (-40.8, -11.7)*** |
| % Change AHI (SE) | -3.5 (9.71) | -57.6 (9.15) | -54.0 (-80.8, -27.3)*** | 3.0 (8.62) | -57.5 (8.27) | -60.5 (-84.5, -36.5)*** | -17.1 (5.92) | -49.3 (5.24) | -32.1 (-48.2, -16.0)*** |
| Change SASHB, %min/h | -9.0 (9.61) | -47.0 (2.93) | -38.0 (-57.7, -18.3) | -11.1 (21.64) | -103.5 (7.47) | -92.4 (-137.2, -47.5) | -74.5 (47.49) | -235.2 (19.06) | -160.7 (-261.0, -60.4) |
| % Change SASHB | -13.9 (14.81) | -72.3 (4.51) | -67.9 (-80.0, -48.4)*** | -7.1 (13.88) | -66.4 (4.79) | -63.8 (-76.1, -45.2)*** | -20.1 (12.84) | -63.6 (5.16) | -54.4 (-70.5, -29.5)*** |
| Change Body Weight, kg | -2.7 (1.49) | -20.7 (1.49) | -18.0 (-22.2, -13.8) | 1.7 (1.54) | -21.4 (1.49) | -23.1 (-27.4, -18.8) | -3.8 (2.01) | -21.0 (1.87) | -17.2 (-22.7, -11.7) |
| % Change body weight | -2.3 (1.26) | -18.4 (1.26) | -16.1 (-19.7, -12.5)*** | 0.9 (1.12) | -18.9 (1.07) | -19.8 (-22.9, -16.7)*** | -3.3 (1.46) | -16.9 (1.34) | -13.6 (-17.6, -9.6)*** |
| Change SBP, mmHg | -1.6 (1.83) | -9.3 (1.77) | -7.7 (-12.8, -2.6)** | -4.6 (1.84) | -9.9 (1.74) | -5.4 (-10.4, -0.3)* | -3.0 (1.71) | -10.1 (1.49) | -7.1 (-11.7, -2.5)** |
| OR achieving ≥50% AHI reduction | 25.1 (8.11) | 71.0 (7.91) | 6.95  (2.23, 21.68)*** | 15.7 (6.45) | 66.2 (8.15) | 10.50  (3.05, 36.16)*** | 15.8 (7.04) | 50.6 (8.78) | 5.04  (1.45, 17.58)* |
| OR achieving AHI <5 or 5-14 with ESS ≤10 | 30.5 (8.30) | 80.5 (7.32) | 8.95  (2.62, 30.55)*** | 8.5 (4.72) | 36.2 (8.13) | 6.10  (1.54, 24.21)* | 0.3 (1.28) | 9.7 (5.17) | 8.72  (0.50, 153.67) |
| Severity Shifts | 38.0 (8.75) | 84.8 (6.55) | 8.4 (2.44, 29.16)*** | 26.6 (7.62) | 74.3 (7.41) | 7.6 (2.50, 22.76)*** | 7.6 (4.99) | 31.8 (8.09) | 5.4 (1.14, 25.96)* |

Note: Data are LSMean (SE), MiTT population, Efficacy Analysis Set. OR = Odds Ratio. * p < 0.05; ** p < 0.01; *** p < 0.001. P-values were not calculated for change in SASHB treatment difference.

Note 2: “Treatment Difference” column refers to Odds Ratio (95% CI), where CI = Confidence interval.

## **TABLE S13:** **Subgroup AHI: Study 2:** Change from baseline in participants receiving treatment in SURMOUNT-OSA in multiple measures and targets at week 52.

| **OSA Outcome** | **Study 2 – On PAP Therapy** | | | | | | | | |
| --- | --- | --- | --- | --- | --- | --- | --- | --- | --- |
|  | **15 - <30** | | | **30 - <70** | | | **≥70** | | |
|  | **PBO** | **TZP** | **Treatment difference** | **PBO** | **TZP** | **Treatment difference** | **PBO** | **TZP** | **Treatment difference** |
| Change AHI, no. of events/h | 4.5 (3.07) | -12.6 (2.84) | -17.1 (-25.5, -8.7)*** | -8.4 (3.38) | -30.9 (2.52) | -22.5 (-30.9, -14.1)*** | -18.2 (5.09) | -52.2 (6.15) | -34.0 (-50.1, -17.8)*** |
| % Change AHI (SE) | 16.0 (13.53) | -55.0 (12.47) | -71.0 (-108.0, -34.0)*** | -16.0 (6.63) | -65.5 (4.95) | -49.5 (-66.0, -33.0)*** | -20.4 (5.90) | -64.0 (7.13) | -43.6 (-62.3, -24.9)*** |
| Change SASHB, %min/h | -9.4 (12.77) | -44.5 (3.65) | -35.0 (-61.1, -9.0) | -34.7 (21.88) | -113.9 (4.57) | -78.9 (-122.7, -35.1) | -142.2 (42.27) | -250.9 (20.24) | -108.7 (-200.6, -16.9) |
| % Change SASHB | -15.7 (21.28) | -74.1 (6.09) | -69.3 (-84.6, -38.5)** | -24.1 (15.16) | -78.7 (3.17) | -72.0 (-82.9, -53.9)*** | -44.3 (13.16) | -78.1 (6.30) | -60.8 (-81.5, -16.7)* |
| Change Body Weight, kg | -1.5 (1.70) | -25.0 (1.65) | -23.5 (-28.2, -18.8) | -3.3 (1.61) | -22.0 (1.21) | -18.8 (-22.8, -14.8) | -3.2 (2.06) | -23.8 (2.70) | -20.5 (-27.3, -13.7) |
| % Change body weight | -1.5 (1.39) | -21.8 (1.35) | -20.3 (-24.2, -16.5)*** | -2.9 (1.26) | -19.2 (0.95) | -16.3 (-19.5, -13.2)*** | -2.7 (1.46) | -19.6 (1.88) | -16.9 (-21.7, -12.2)*** |
| Change SBP, mmHg | -2.4 (1.61) | -9.8 (1.49) | -7.4 (-11.8, -3.0)** | -0.9 (2.15) | -8.3 (1.58) | -7.4 (-12.8, -2.1)** | -0.8 (2.03) | -8.3 (2.45) | -7.5 (-13.9, -1.1)* |
| OR achieving ≥50% AHI reduction | 23.1 (8.00) | 74.2 (7.81) | 9.00  (2.62, 30.98)*** | 29.1 (8.09) | 75.5 (5.79) | 7.06  (2.64, 18.84)*** | 17.0 (6.51) | 71.1 (10.38) | 10.08  (2.77, 36.70)*** |
| OR achieving AHI <5 or 5-14 with ESS ≤10 | 26.9 (8.23) | 73.9 (7.85) | 7.09  (2.15, 23.31)** | 11.1 (5.88) | 48.1 (6.58) | 7.00  (2.02, 24.22)** | 2.6 (2.58) | 24.2 (9.45) | 10.17  (1.37, 75.32)* |
| Severity Shifts | 30.1 (8.63) | 81.4 (6.81) | 9.5 (2.72, 33.26)*** | 34.1 (8.37) | 79.8 (5.37) | 7.5 (2.76, 20.25)*** | 9.3 (4.99) | 64.6 (10.86) | 14.1 (3.43, 57.77)*** |

Note: Data are LSMean (SE), MiTT population, Efficacy Analysis Set. PAP = Positive Airway Pressure; AHI = apnea-hypoapnea index; SASHB = sleep apnea-specific hypoxic burden (total hypoxic burden); BW = Body Weight; SBP = Systolic Blood Pressure; OR = Odds Ratio. * p < 0.05; ** p < 0.01; *** p < 0.001. P-values were not calculated for change in SASHB treatment difference.

Note 2: “Treatment Difference” column refers to Odds Ratio (95% CI), where CI = Confidence interval.

## **TABLE S14:** **Subgroup BMI: Study 1:** Change from baseline in participants receiving treatment in SURMOUNT-OSA in multiple measures and targets at week 52.

| **OSA Outcome** | **Study 1 – Not on PAP Therapy** | | | | | | | | |
| --- | --- | --- | --- | --- | --- | --- | --- | --- | --- |
|  | **BMI group 30 - <35** | | | **BMI group ≥35 - <40** | | | **BMI group ≥40** | | |
|  | **PBO** | **TZP** | **Treatment difference** | **PBO** | **TZP** | **Treatment difference** | **PBO** | **TZP** | **Treatment difference** |
| Change in AHI (SE), no. of events/h | -6.2 (3.67) | -25.2 (3.49) | -19.0 (-29.2, -8.8)*** | -5.7 (3.35) | -29.2 (3.11) | -23.5 (-32.6, -14.3)*** | -1.4 (4.77) | -27.9 (4.31) | -26.4 (-39.3, -13.5)*** |
| % Change AHI (SE) | -2.4 (9.16) | -56.2 (8.62) | -53.7 (-79.0, -28.4)*** | -13.9 (6.28) | -60.6 (5.83) | -46.7 (-63.9, -29.5)*** | 4.5 (9.55) | -49.9 (8.68) | -54.4, (-80.3, -28.5)*** |
| Change SASHB, %min/h | -7.0 (21.98) | -95.8 (7.14) | -88.7 (-134.0, -43.4) | -20.8 (18.15) | -103.5 (5.46) | -82.7 (-119.9, -45.6) | -33.0 (23.76) | -107.5 (10.04) | -74.4 (-125.0, -23.9) |
| % Change SASHB | -4.9 (15.38) | -67.0 (5.00) | -65.3 (-77.8, -45.8)*** | -14.5 (12.67) | -72.2 (3.81) | -67.5 (-78.3, -51.3)*** | -19.2 (13.79) | -62.4 (5.83) | -53.5 (-70.7, -26.1)** |
| Change Body Weight, kg | -0.7 (1.05) | -16.3 (1.09) | -15.6 (-18.6, -12.5)*** | -2.5 (1.20) | -22.5 (1.12) | -20.0 (-23.2, -16.7)*** | -0.9 (2.23) | -23.7 (2.07) | -22.8 (-28.8, -16.7)*** |
| % Change Body Weight | -0.7 (1.01) | -17.2 (1.02) | -16.5 (-19.3, -13.6)*** | -2.4 (1.03) | -20.2 (0.96) | -17.8 (-20.7, -15.0)*** | -1.0 (1.58) | -17.0 (1.46) | -16.0 (-20.3, -11.7)*** |
| Change SBP, mmHg | -1.7 (1.64) | -9.3 (1.57) | -7.6 (-12.2, -3.0)** | -2.3 (1.87) | -10.0 (1.72) | -7.7 (-12.7, -2.6)** | -3.7 (1.93) | -9.6 (1.76) | -5.9 (-11.2, -0.7)* |
| OR achieving ≥50% AHI reduction | 21.8 (7.55) | 65.2 (8.67) | 6.9 (2.13. 22.48)** | 14.8 (6.74) | 67.6 (8.06) | 11.9 (3.28, 43.29)*** | 20.2 (7.05) | 55.1 (8.40) | 4.8 (1.58, 14.89)** |
| OR achieving AHI <5 or 5-14 with ESS ≤10 | 13.6 (6.52) | 48.2 (9.10) | 18.8 (2.7, 131.7)** | 14.5 (6.41) | 53.3 (8.58) | 10.1 (2.33, 43.45)** | 16.7 (6.49) | 29.5 (7.40) | 3.1 (0.68, 14.18) |
| Severity Shifts | 23.9 (7.88) | 65.9 (8.58) | 8.02 (2.23, 28.87)** | 32.1 (8.43) | 70.2 (7.96) | 7.48 (1.91, 29.35)** | 20.7 (7.09) | 57.02 (8.16) | 8.59 (2.29, 32.20)** |

Note: Data are LSMean (SE), MiTT population, Efficacy Analysis Set. PAP = Positive Airway Pressure; AHI = apnea-hypoapnea index; SASHB = sleep apnea-specific hypoxic burden (total hypoxic burden); BW = Body Weight; SBP = Systolic Blood Pressure; OR = Odds Ratio * p < 0.05; ** p < 0.01; *** p < 0.001 vs Placebo. P-values were not calculated for change in SASHB treatment difference.

Note 2: “Treatment Difference” column refers to Odds Ratio (95% CI), where CI = Confidence interval.

## **TABLE S15:** **Subgroup BMI: Study 2:** Change from baseline in participants receiving treatment in SURMOUNT-OSA in multiple measures and targets at week 52.

| **OSA Outcome** | **Study 2 – On PAP Therapy** | | | | | | | | |
| --- | --- | --- | --- | --- | --- | --- | --- | --- | --- |
|  | **BMI group 30 - <35** | | | **BMI group ≥35 - <40** | | | **BMI group ≥40** | | |
|  | **PBO** | **TZP** | **Treatment difference** | **PBO** | **TZP** | **Treatment difference** | **PBO** | **TZP** | **Treatment difference** |
| Change in AHI (SE), no. of events/h | -7.4 (3.54) | -30.3 (3.35) | -23.0 (-32.9, -13.0)*** | -6.6 (4.04) | -34.4 (3.49) | -27.8 (-38.5, -17.1)*** | -4.2 (3.91) | -26.0 (3.47) | -21.8 (-32.4, -11.2)*** |
| % Change AHI (SE) | -13.3 (10.02) | -60.4 (9.49) | -47.1 (-75.2, -19.0)*** | -8.3 (7.84) | -67.4 (6.73) | -59.2 (-79.9, -38.4)*** | 3.1 (10.38) | -59.4 (9.19) | -62.4 (-90.4, -34.5)*** |
| Change SASHB, %min/h | -35.3 (19.02) | -102.6 (4.79) | -67.3 (-105.8, -28.9) | -61.2 (24.80) | -119.4 (7.00) | -58.3 (-108.8, -7.8) | -25.9 (15.02) | -84.3 (5.62) | -58.4 (-89.8, -27.0) |
| % Change SASHB | -27.8 (14.98) | -80.8 (3.77) | -73.4 (-85.1, -52.6)*** | -41.4 (16.76) | -80.7 (4.73) | -67.2 (-84.6, -29.8) ** | -20.5 (11.93) | -66.9 (4.46) | -58.4 (-72.2, -37.6)*** |
| Change Body Weight, kg | -0.8 (1.26) | -18.7 (1.23) | -17.8 (-21.4, -14.3)*** | -3.7 (1.43) | -23.0 (1.27) | -19.2 (-23.0, -15.4)*** | -2.8 (2.34) | -27.8 (2.20) | -25.0 (-31.5, -18.6)*** |
| % Change Body Weight | -1.1 (1.25) | -19.0 (1.22) | -17.9 (-21.4, -14.4)*** | -3.2 (1.21) | -20.5 (1.08) | -17.3 (-20.5, -14.1)*** | -2.2 (1.61) | -20.8 (1.51) | -18.6 (-23.0, -14.2)*** |
| Change SBP, mmHg | -0.5 (1.60) | -9.7 (1.52) | -9.2 (-13.7, -4.7)*** | -1.7 (1.90) | -6.7 (1.60) | -5.0 (-10.0, 0.0)* | -2.8 (2.39) | -10.2 (2.15) | -7.4 (-13.9, -0.9)* |
| OR achieving ≥50% AHI reduction | 27.0 (8.17) | 74.8 (7.95) | 6.9 (2.08, 23.14)** | 24.4 (7.69) | 80.3 (6.24) | 11.1 (3.66, 33.44)*** | 18.0 (6.77) | 66.8 (7.89) | 7.3 (2.32, 23.19)*** |
| OR achieving AHI <5 or 5-14 with ESS ≤10 | 17.4 (7.0) | 56.7 (8.87) | 7.6 (1.92, 30.46)** | 15.3 (6.53) | 46.2 (7.70) | 6.4 (1.63, 24.80)** | 8.7 (5.07) | 53.5 (8.27) | 10.0 (2.30, 43.19)** |
| Severity Shifts | 30.2 (8.42) | 79.1 (7.41) | 8.79 (2.45, 31.57)*** | 22.6 (7.55) | 80.5 (6.14) | 14.40 (4.39, 47.23)*** | 21.03 (7.01) | 72.7 (7.39) | 7.43 (2.40, 23.06)*** |

Note 1: Data are LSMean (SE), MiTT population, Efficacy Analysis Set. PAP = Positive Airway Pressure; AHI = apnea-hypoapnea index; SASHB = sleep apnea-specific hypoxic burden (total hypoxic burden); BW = Body Weight; SBP = Systolic Blood Pressure; OR = Odds Ratio. * p < 0.05; ** p < 0.01; *** p < 0.001 vs Placebo. P-values were not calculated for change in SASHB treatment difference.

Note 2: “Treatment Difference” column refers to Odds Ratio (95% CI), where CI = Confidence interval.

## **TABLE S16:** **Subgroup Neck Circumference: Study 1:** Change from baseline in participants receiving treatment in SURMOUNT-OSA in multiple measures and targets at week 52.

| **OSA Outcome** | **Study 1 – Not on PAP Therapy** | | | | | |
| --- | --- | --- | --- | --- | --- | --- |
|  | **Small NC** | | | **Large NC** | | |
|  | **PBO** | **TZP** | **Treatment difference** | **PBO** | **TZP** | **Treatment difference** |
| Change in AHI (SE), no. of events/h | -5.4 (3.27) | -23.9 (3.24) | -18.5 (-27.7, -9.3)*** | -3.7 (3.21) | -30.5 (2.84) | -26.9 (-35.4, -18.3)*** |
| % Change AHI (SE) | -7.3 (7.9) | -51.0 (7.75) | -43.8 (-65.9, -21.7)*** | -2.5 (5.95) | -58.5 (5.27) | -56.0 (-71.8, -40.1)*** |
| Change SASHB, %min/h | -21.9 (14.65) | -85.5 (5.99) | -63.6 (-94.7, -32.6) | -18.0 (18.94) | -117.6 (5.70) | -99.6 (-138.4, -60.9) |
| % Change SASHB | -16.7 (11.20) | -65.4 (4.58) | -58.4 (-71.5, -39.4)*** | -10.6 (11.18) | -69.5 (3.37) | -65.8 (-75.5, -52.4)*** |
| Change Body Weight, kg | -1.3 (1.15) | -17.1 (1.19) | -15.7 (-19.0, -12.5)*** | -1.4 (1.42) | -23.7 (1.29) | -22.3 (-26.1, -18.5)*** |
| % Change Body Weight | -1.3 (1.03) | -17.1 (1.06) | -15.8 (-18.7, -12.8)*** | -1.5 (1.04) | -18.8 (0.94) | -17.3 (-20.0, -14.5)*** |
| Change SBP, mmHg | -2.3 (1.51) | -9.0 (1.48) | -6.7 (-11.0, -2.5)** | -3.3 (1.46) | -10.0 (1.29) | -6.7 (-10.6, -2.8)*** |
| OR achieving ≥50% AHI reduction | 22.9 (6.56) | 68.6 (7.30) | 6.88 (2.51, 18.86)*** | 16.6 (5.71) | 59.3 (6.40) | 8.80 (3.12, 24.82)*** |
| OR achieving AHI <5 or 5-14 with ESS ≤10 | 12.7 (5.44) | 50.2 (8.02) | 10.95 (2.65, 45.22)*** | 16.1 (5.14) | 38.8 (6.22) | 6.82 (2.03, 22.89)** |
| Severity Shifts | 26.7 (6.80) | 71.4 (7.10) | 6.96 (2.50, 19.35)*** | 24.9 (6.11) | 60.4 (6.28) | 10.50 (3.13, 35.14)*** |

Note 1: Data are LSMean (SD), MITT population, Efficacy Analysis Set. Large NC cut off for female is ≥41 cm and for male is ≥43 cm. PAP = Positive Airway Pressure; AHI = apnea-hypoapnea index; SASHB = sleep apnea-specific hypoxic burden (total hypoxic burden); BW = Body Weight; SBP = Systolic Blood Pressure; OR = Odds Ratio. * p < 0.05; ** p < 0.01; *** p < 0.001 vs Placebo. P-values were not calculated for change in SASHB treatment difference.

Note 2: “Treatment Difference” column refers to Odds Ratio (95% CI), where CI = Confidence interval.

## **TABLE S17:** **Subgroup Neck Circumference: Study 2:** Change from baseline in participants receiving treatment in SURMOUNT-OSA in multiple measures and targets at week 52.

| **OSA Outcome** | **Study 2 – On PAP Therapy** | | | | | |
| --- | --- | --- | --- | --- | --- | --- |
|  | **Small NC** | | | **Large NC** | | |
|  | **PBO** | **TZP** | **Treatment difference** | **PBO** | **TZP** | **Treatment difference** |
| Change in AHI (SE), no. of events/h | -1.9 (4.16) | -29.5 (3.4) | -27.6 (-38.5, -16.7)*** | -7.4 (2.63) | -30.8 (2.47) | -23.4 (-30.7, -16.2)*** |
| % Change AHI (SE) | -3.9 (11.68) | -59.0 (9.40) | -55.1 (-85.5, -24.8)*** | -7.8 (5.97) | -64.6 (5.60) | -56.8 (-73.1, -40.5)*** |
| Change SASHB, %min/h | -28.3 (25.01) | -89.0 (5.27) | -60.7 (-110.7, -10.6) | -46.6 (13.46) | -109.3 (4.69) | -62.7 (-90.7, -34.8) |
| % Change SASHB | -25.7 (22.70) | -80.8 (4.78) | -74.1 (-88.3, -42.6)*** | -31.9 (9.21) | -74.8 (3.21) | -63.0 (-74.4, -46.5)*** |
| Change Body Weight, kg | -2.8 (1.43) | -20.6 (1.22) | -17.9 (-21.6, -14.1)*** | -2.6 (1.32) | -24.5 (1.28) | -21.9 (-25.6, -18.3)*** |
| % Change Body Weight | -3.0 (1.30) | -20.1 (1.10) | -17.1 (-20.5, -13.7)*** | -2.1 (0.97) | -20.1 (0.94) | -18.0 (-20.7, -15.4)*** |
| Change SBP, mmHg | -2.2 (1.86) | -7.4 (1.51) | -5.1 (-10.0, -0.2)* | -1.5 (1.41) | -9.4 (1.32) | -7.9 (-11.7, -4.1)*** |
| OR achieving ≥50% AHI reduction | 27.1 (8.76) | 74.6 (7.27) | 7.14 (2.23, 22.85)*** | 21.4 (5.13) | 74.2 (5.13) | 9.35 (4.19, 20.85)*** |
| OR achieving AHI <5 or 5-14 with ESS ≤10 | 23.8 (8.32) | 51.4 (8.26) | 5.07 (1.46, 17.59)* | 9.9 (3.90) | 51.5 (5.82) | 10.83 (3.63, 32.29)*** |
| Severity Shifts | 34.8 (9.31) | 80.1 (6.69) | 8.06 (2.40, 27.06)*** | 20.5 (4.98) | 76.4 (4.94) | 10.95 (4.74, 25.29)*** |

Note 1: Data are LSMean (SD), MITT population, Efficacy Analysis Set. Large NC cut off for female is >=16 inch (40.64 cm) and for male is >=17 inch (43.18 cm) using a conversion rate of 2.54. PAP = Positive Airway Pressure; AHI = apnea-hypoapnea index; SASHB = sleep apnea-specific hypoxic burden (total hypoxic burden); BW = Body Weight; SBP = Systolic Blood Pressure; OR = Odds Ratio. * p < 0.05; ** p < 0.01; *** p < 0.001 vs Placebo. P-values were not calculated for change in SASHB treatment difference.

Note 2: “Treatment Difference” column refers to Odds Ratio (95% CI), where CI = Confidence interval.

## **TABLE S18:** **Summary of missing data**

| **Variable** | **Study 1** | | **Study 2** | | |
| --- | --- | --- | --- | --- | --- |
|  | **PBO (N=120)** | **TZP (N=114)** | **PBO (N=114)** | **TZP (N=119)** |  |
| **Sex** |  |  |  |  |  |
| Baseline, n | 0 | 0 | 0 | 0 |  |
| **Age** |  |  |  |  |  |
| Baseline, n | 0 | 0 | 0 | 0 |  |
| **AHI** |  |  |  |  |  |
| Baseline, n | 1 | 0 | 0 | 0 |  |
| **BMI** |  |  |  |  |  |
| Baseline, n | 1 | 2 | 0 | 2 |  |
| **Hypoxic burden (OSA risk)** |  |  |  |  |  |
| Baseline, n | 2 | 1 | 0 | 0 |  |

# **Table S19: Study Disposition by Baseline Subgroup**

| **Variable** | **Study 1** | | | | **Study 2** | | | | |
| --- | --- | --- | --- | --- | --- | --- | --- | --- | --- |
|  | **PBO** | | **TZP** | | **PBO** | | **TZP** | | |
|  | **Completed** | **Discontinued** | **Completed** | **Discontinued** | **Completed** | **Discontinued** | **Completed** | | **Discontinued** |
| **Age <50** | 42 (67.7) | 20 (32.3) | 60 (95.2) | 3 (4.8) | 35 (77.8) | 10 (22.2) | 49 (92.5) | | 4 (7.5) |
| **Age ≥50** | 44 (75.9) | 14 (24.1) | 41 (80.4) | 10 (19.6) | 54 (78.3) | 15 (21.7) | 64 (97.0) | | 2 (3.0) |
| **Female** | 31 (75.6) | 10 (24.4) | 31 (86.1) | 5 (13.9) | 26 (81.2) | 6 (18.8) | 32 (100.0) | | 0 |
| **Male** | 55 (69.6) | 24 (30.4) | 70 (89.7) | 8 (10.3) | 63 (76.8) | 19 (23.2) | 81 (93.1) | | 6 (6.9) |
| **AHI <30 events/h** | 32 (69.6) | 14 (30.4) | 34 (85.0) | 6 (15.0) | 28 (71.8) | 11 (28.2) | 35 (100.0) | 0 | |
| **AHI 30-70 events/h** | 31 (79.5) | 8 (20.5) | 36 (94.7) | 2 (5.3) | 31 (86.1) | 5 (13.9) | 59 (93.7) | 4 (6.3) | |
| **AHI ≥70 events/h** | 23 (67.6) | 11 (32.4) | 31 (86.1) | 5 (13.9) | 30 (76.9) | 9 (23.1) | 19 (90.5) | 2 (9.5) | |
| **BMI 30 to < 35 kg/m^2^** | 29 (67.4) | 14 (32.6) | 28 (90.3) | 3 (9.7) | 28 (84.8) | 5 (15.2) | 31 (100.0) | 0 | |
| **BMI 35 to <40 kg/m^2^** | 28 (80.0) | 7 (20.0) | 35 (89.7) | 4 (10.3) | 31 (75.6) | 10 (24.4) | 44 (93.6) | 3 (6.4) | |
| **BMI ≥40 kg/m^2^** | 28 (68.3) | 13 (31.7) | 36 (85.7) | 6 (14.3) | 30 (75.0) | 10 (25.0) | 37 (94.9) | 2 (5.1) | |
| **Small NC** | 40 (78.4) | 11 (21.6) | 40 (90.9) | 4 (9.1) | 24 (80.0) | 6 (20.0) | 36 (94.7) | 2 (5.3) | |
| **Large NC** | 45 (67.2) | 22 (32.8) | 61 (88.4) | 8 (11.6) | 65 (77.4) | 19 (22.6) | 77 (95.1) | 4 (4.9) | |

# **Table S20: Treatment Disposition by Baseline Subgroup**

| **Variable** | **Study 1** | | | | **Study 2** | | | | |
| --- | --- | --- | --- | --- | --- | --- | --- | --- | --- |
|  | **PBO** | | **TZP** | | **PBO** | | **TZP** | | |
|  | **Completed** | **Discontinued** | **Completed** | **Discontinued** | **Completed** | **Discontinued** | **Completed** | | **Discontinued** |
| **Age <50** | 40 (64.5) | 22 (35.5) | 57 (90.5) | 6 (9.5) | 32 (71.1) | 13 (28.9) | 49 (92.5) | | 4 (7.5) |
| **Age ≥50** | 44 (75.9) | 14 (24.1) | 40 (78.4) | 11 (21.6) | 53 (76.8) | 16 (23.2) | 59 (89.4) | | 7 (10.6) |
| **Female** | 30 (73.2) | 11 (26.8) | 30 (83.3) | 6 (16.7) | 25 (78.1) | 7 (21.9) | 30 (93.8) | | 2 (6.2) |
| **Male** | 54 (68.4) | 25 (31.6) | 67 (85.9) | 11 (14.1) | 60 (73.2) | 22 (26.8) | 78 (89.7) | | 9 (10.3) |
| **AHI <30 events/h** | 30 (65.2) | 16 (34.8) | 32 (80.0) | 8 (20.0) | 28 (71.8) | 11 (28.2) | 33 (94.3) | 2 (5.7) | |
| **AHI 30-70 events/h** | 31 (79.5) | 8 (20.5) | 35 (92.1) | 3 (7.9) | 30 (83.3) | 6 (16.7) | 56 (88.9) | 7 (11.1) | |
| **AHI ≥70 events/h** | 23 (67.6) | 11 (32.4) | 30 (83.3) | 6 (16.7) | 27 (69.2) | 12 (30.8) | 19 (90.5) | 2 (9.5) | |
| **BMI 30 to < 35 kg/m^2^** | 27 (62.8) | 16 (37.2) | 28 (90.3) | 3 (9.7) | 27 (81.8) | 6 (18.2) | 30 (96.8) | 1 (3.2) | |
| **BMI 35 to <40 kg/m^2^** | 28 (80.0) | 7 (20.0) | 33 (84.6) | 6 (15.4) | 30 (73.2) | 11 (26.8) | 42 (89.4) | 5 (10.6) | |
| **BMI ≥40 kg/m^2^** | 28 (68.3) | 13 (31.7) | 34 (81.0) | 8 (19.0) | 28 (70.0) | 12 (30.0) | 35 (89.7) | 4 (10.3) | |
| **Small NC** | 39 (76.5) | 12 (23.5) | 40 (90.9) | 4 (9.1) | 23 (76.7) | 7 (23.3) | 36 (94.7) | 2 (5.3) | |
| **Large NC** | 44 (65.7) | 23 (34.3) | 57 (82.6) | 12 (17.4) | 62 (73.8) | 22 (26.2) | 72 (88.9) | 9 (11.1) | |

# SUPPLEMENTARY FIGURES

## FIGURE S1: % participants with improvement by subgroup Age (PBO and TZP arms)

## FIGURE S2: % participants with improvement by subgroup Sex (PBO and TZP arms)

## FIGURE S3: % participants with improvement by subgroup AHI (PBO and TZP arms)

## FIGURE S4: % participants with improvement by subgroup BMI (PBO and TZP arms)

## FIGURE S5: % participants with improvement by subgroup Neck Circumference (PBO and TZP arms)

# References

1.	Chang, J.L., et al., *International Consensus Statement on Obstructive Sleep Apnea.* Int Forum Allergy Rhinol, 2023. **13**(7): p. 1061-1482.
